# BRCA1 Deficiency Impairs Mitophagy and Promotes Inflammasome Activation and Mammary Tumor Metastasis

**DOI:** 10.1002/advs.201903616

**Published:** 2020-02-14

**Authors:** Qiang Chen, Josh Haipeng Lei, Jiaolin Bao, Haitao Wang, Wenhui Hao, Licen Li, Cheng Peng, Takaaki Masuda, Kai Miao, Jun Xu, Xiaoling Xu, Chu‐Xia Deng

**Affiliations:** ^1^ Cancer Center Faculty of Health Sciences University of Macau Macau Macau, SAR China; ^2^ Center for Precision Medicine Research and Training University of Macau Macau Macau, SAR China; ^3^ Zhuhai UM Science and Technology Research Institute Zhuhai 519031 China; ^4^ Department of Surgery Kyushu University Beppu Hospital Beppu‐shi Oita 874‐0838 Japan

**Keywords:** ATM‐AMPK‐DRP1, BRCA1, inflammasomes, mitochondrial dynamics, mitophagy

## Abstract

The breast cancer susceptibility gene 1 (*BRCA1*) is a major tumor suppressor gene and is most frequently mutated in hereditary breast cancer. BRCA1 plays a critical role in many biological processes, especially maintaining genomic stability in the nucleus, yet its role in the cytoplasm remains elusive. Here, it is revealed that BRCA1 maintains a healthy mitochondrial network through regulating mitochondrial dynamics, including fission and fusion. BRCA1 deficiency causes dysfunctional mitochondrial dynamics through increased expression of mitofusin1/2. With mitochondrial stress, BRCA1 is recruited to the mitochondrial outer membrane, where it plays an essential role in maintaining a healthy mitochondrial network. Consequently, BRCA1 deficiency impairs stress‐induced mitophagy through blocking ataxia‐telangiectasia mutated (ATM)‐AMP‐activated protein kinase (AMPK)‐Dynamin‐related protein 1 (DRP1)‐mediated mitochondrial fission and triggers NLRP3 inflammasome activation, which creates a tumor‐associated microenvironment, thereby facilitating tumor proliferation and metastasis. It is further shown that inflammasome inhibition can prevent tumor recurrence and metastasis. This study uncovers an important role of BRCA1 in regulating mitophagy and suggests a therapeutic approach for fighting this deadly disease.

## Graphical abstract

Breast cancer susceptibility gene 1 (BRCA1) not only negatively regulates mitochondrial fusion but also facilitates stress‐induced fission and mitophagy. BRCA1 deficiency causes oxidative stress and inflammasome activation, which creates a tumor‐associated microenvironment, thereby promoting breast cancer development. This study uncovers the function of BRCA1 in regulating mitophagy and provides a rational approach for treating *BRCA1* mutant breast cancer with inflammasome inhibitors.

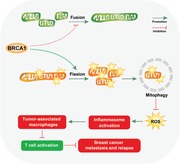

## 1 Introduction

Breast cancer is the most common disease in women, which accounts for 30% of all new cancers.[qv: 1] The clinical and molecular heterogeneity of breast cancer is well known. Based on molecular classification, triple‐negative breast cancer (TNBC), which lacks expression of estrogen receptor (ER), progesterone receptor (PR), and Her2, accounts for ≈12–17% of breast cancer.[qv: 2] Patients with TNBC have a relatively poorer prognosis compared with those with other breast cancer subtypes; this is due to its aggressive clinical properties and lack of established molecular targets for therapy.[qv: 3] Therefore, there has been an intense interest in finding new medications that can treat TNBC.

The breast cancer susceptibility gene 1 (*BRCA1*) is a major breast cancer suppressor gene, which encodes a protein critical for maintaining DNA integrity and genomic stability. More than 75% of tumors developing in women who carry *BRCA1* mutations are TNBC.[qv: 4] BRCA1 tumor suppressor activity has been attributed to its nuclear localization, where it participates in signaling pathways for DNA damage repair, transcription regulation, chromatin remodeling, cell cycle checkpoint control, and apoptosis.[qv: 5,6] Meanwhile, BRCA1 has been identified as a protein that shuttles between the nucleus and the cytoplasm.[qv: 7] Nuclear export of BRCA1 could be induced by DNA damage in the p53 dependent mechanism.[qv: 8] However, the function of BRCA1 in cytoplasmic processes, which may be independent from maintenance of genomic stability, is poorly understood.

Mitochondria are crucial organelles for energy production and cellular homeostasis in mammalian cells; therefore, the maintenance of a healthy mitochondrial network is critical in the development as well as in the response to physiological adaptations and stress conditions throughout life.[qv: 9] Mitophagy, a selective autophagic process, plays an important role in maintaining mitochondrial function. Mitochondria as dynamic organelles are constantly undergoing fission and fusion, which are essential for regulation of mitophagy.[qv: 10] Defects in mitophagy could lead to pathological conditions, such as neurodegeneration, inflammasome activation, and cancer.[qv: 11–13] Recent studies indicated that BRCA1 deficiency could impair oxidative phosphorylation and decrease ATP production in cardiac and muscle tissues,[qv: 14–16] suggesting that BRCA1 is involved in mitochondrial functions. However, little is known about how BRCA1 relates to mitophagy in response to mitochondrial damage and how defects in mitophagy contribute to BRCA1‐associated breast cancer.

In this study, we seek to determine the mechanism by which BRCA1 is involved in mitophagy and its impact on therapeutic treatment of BRCA1‐associated breast cancer. Our findings demonstrate that BRCA1 deficiency impairs mitochondrial function and mitophagy through AMP‐activated protein kinase (AMPK)‐mediated mitochondrial fission and induces inflammasome activation, which then promotes metastasis of *Brca1* mutant mammary tumor. This suggests that inflammasome inhibition could serve as a therapeutic target for the treatment of BRCA1‐associated breast cancer.

## 2 Results

### 2.1 BRCA1 Is Essential for Mitophagy

**Figure 1:**
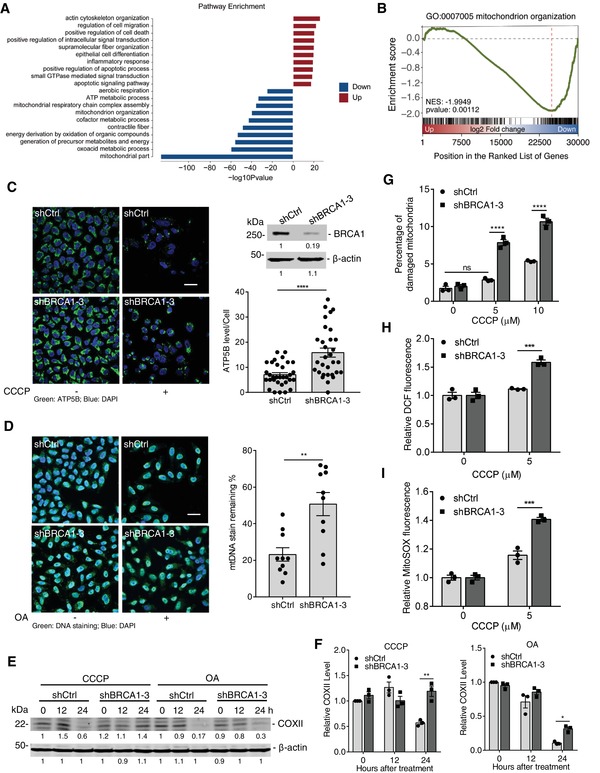
BRCA1 is required for stress‐induced mitophagy. A) Top pathways from GSEA upregulated and downregulated genes in *Brca1* MT versus WT mammary glands by using gene ontology (GO) analysis. B) GSEA plot of enrichment in “mitochondrion organization” gene set, significantly downregulated in *Brca1* MT mammary glands. C) Analysis of mitophagy activity in shCtrl and shBRCA1 Hela‐mCherryParkin under CCCP treatment by clearance of ATP5B. Left panel: Representative images of cells immunostained to ATP5B (green); DAPI, DNA‐binding dye; Scale bar, 20 µm. Right upper panel: Western blot for BRCA1. Right lower panel: Quantification for ATP5B level after CCCP treatment (more than 30 cells were counted per group). D) Representative images of shCtrl and shBRCA1 cells immunostained to mtDNA in the absence and presence of OA treatment (left panel, scale bar, 20 µm) and quantified for mitophagy (right panel) (ten fields counted per group). E) Immunoblot analysis of COXII and β‐actin (loading control throughout) in shCtrl and shBRCA1 cells exposed to CCCP or oligomycin/antimycin A (OA) treatment. F) Quantification of COXII level in (E), which normalized by β‐actin level (*n* = 3 per group). G) Analysis of damaged mitochondria in shCtrl and shBRCA1 MDA‐MB‐231 cells after different dosages of CCCP treatment (*n* = 3 per group). H) Measurement of cytoplasmic and I) mitochondrial ROS in shCtrl and shBRCA1 MDA‐MB‐231 cells treated by CCCP (*n* = 3 per group). Data represent the mean ± SEM and are representative of three independent experiments. Significant differences were determined by an unpaired two‐tailed *t*‐test (C and D) or two‐way ANOVA with Tukey's multiple comparison test (F–I). *p < 0.05, **p < 0.01, ***p < 0.001, ****p < 0.0001.

We previously demonstrated that mice with mammary gland (MG)‐specific deletion of *Brca1* exon 11 (*Brca1^flox/flox^; MMTV‐Cre*) spontaneously developed mammary tumors.[qv: 17] To explore the function of BRCA1 in cytoplasm, we carried out a genome‐wide unbiased approach to analyze gene expression by RNA sequencing (RNA‐Seq) in both *Brca1* mutant (MT) and wild‐type (WT) MGs from *Brca1^flox/flox^; MMTV‐Cre*, and *Brca1^flox/flox^* mice, respectively. Bioinformatics analysis of the whole transcriptome indicates that loss of BRCA1 has a profound impact on gene expression networks related to mitochondrial functions (**Figure** **1**A). Gene set enrichment analysis (GSEA), using the curated gene set compilation hallmark of transcripts downregulated in the MG of MT mice compared with WT mice, detected enriched genes corresponding to mitochondrial organization (Figure 1B). This finding suggests that *BRCA1* mutation might cause dysfunction of mitochondria.

Mitophagy is a specialized autophagy for clearing damaged mitochondria, and plays a critical role in maintaining mitochondrial functions. A major trigger for mitophagy is via the PTEN‐induced putative kinase 1 (PINK1)/Parkin pathway.[qv: 9] To verify the effect of BRCA1 on mitophagy, we first monitored occurrence of mitophagy in Hela cells that express mCherry‐Parkin (Hela‐mCherryParkin cells) with mitochondrial damage treatment in different ways. We performed knockdown (KD) of BRCA1 in Hela‐mCherryParkin cells infected with three different lentivirus‐shRNA against BRCA1 (shBRCA1) and found that shBRCA1–2 and shBRCA1–3 worked well and could block Parkin‐mediated mitophagy after treatment with a chemical mitochondrial uncoupler, carbonyl cyanide 3‐chlorophenylhydrazone (CCCP), as measured by quantitation of the ATP5B level (Figure 1C and Figure S1A–C, Supporting Information). To further address the function of BRCA1 in mitophagy, we used shBRCA1–3 to silence BRCA1 expression for the following experiments. As another indicator of mitophagy, mitochondrial DNA (mtDNA) contents were measured by immunofluorescence. As shown in Figure 1D, mtDNA was eliminated in shCtrl cells after treatment with mitochondrial respiration inhibitors, oligomycin and antimycin A (OA), whereas BRCA1 KD cells contained more mtDNA, indicating an inhibition in mitophagy. Meanwhile, we analyzed mitophagy in shCtrl and BRCA1 KD cells by measuring degradation of mitochondrial inner membrane protein cytochrome C oxidase subunit II (COXII) after treatment with CCCP or OA. The results showed that COXII was more degraded in shCtrl cells compared to BRCA1 KD cells (Figure 1E,F), suggesting that BRCA1 is required for mitophagy.

To further confirm that BRCA1 is also essential for mitophagy in cells with endogenous Parkin, we detected mitophagy in *Brca1^flox/flox^* mouse embryonic fibroblasts (MEFs), infected with adenovirus‐green fluorescent protein (GFP) (AdGFP) or AdCre. These results showed that deletion of *Brca1* impaired mitophagy through measurement of mitochondrial proteins such as COXII, COXIV, and TOM20 (Figure S1D–F, Supporting Information). In addition, we also used the mt‐mKeima assay, which was developed recently for detecting mitophagy activity using a pH‐sensitive fluorescent protein.[qv: 18,19] mt‐mKeima changes excitation peak from green (488 nm) to red (561 nm) when engulfed in lysosomes, which allows assessment of mitophagy.[qv: 18] CCCP‐induced mitophagy was analyzed in *Brca1^flox/flox^* MEFs with tamoxifen‐inducible Cre (*Tam‐Cre*), in which 4‐hydroxytamoxifen (4‐HT) treatment caused BRCA1 deletion (Figure S1G, Supporting Information). The results showed that CCCP significantly resulted in spectral shift of Ctrl group, but had no effect on 4‐HT group, which was reflected by 561/488 ratio of mt‐mKeima (Figure S1H,I, Supporting Information). These results further confirmed that BRCA1 plays the vital role in mitophagy.

### 2.2 Loss of BRCA1 Leads to Accumulation of Damaged Mitochondria via the Blocking of Mitophagy

As indicated above, BRCA1 deficiency impairs mitophagy, which suggests that more damaged mitochondria accumulate in *BRCA1* mutant breast cancer cells under mitochondrial stress. To verify this, the damaged mitochondrial level was monitored in MDA‐MB‐231 cells by measuring mitochondrial membrane potential. The results showed that loss of BRCA1 caused more damaged mitochondria accumulated in cells even under a low dosage of CCCP (5 × 10^−6^ m), which had no obvious effect on shCtrl cells (Figure 1G). Furthermore, shBRCA1 cells produced excessive cytoplasmic and mitochondrial reactive oxygen species (ROS), indicating accumulation of damaged mitochondria after damage from CCCP (Figure 1H,I). The results demonstrate that *Brca1* deletion causes accumulation of damaged mitochondria through inhibiting mitophagy, and the loss of BRCA1 impairs mitochondrion‐related signaling pathways in the mammary gland (Figure 1A,B).

### 2.3 BRCA1 Is Essential for Autophagosome Formation during Mitophagy

The initiation of mitophagy is mediated by the stabilization of PINK1 and translocation of Parkin to the mitochondria.[qv: 20] To address how BRCA1 regulates mitophagy with mitochondrial damage, we first evaluated whether BRCA1 mediated activation of the PINK1/Parkin pathway. As shown in Figure S2A,B in the Supporting Information, CCCP treatment induced accumulation of PINK1 and mitochondrial recruitment of Parkin in both shCtrl cells and shBRCA1 cells, suggesting that BRCA1 is not involved in PINK1/Parkin activation. Parkin, as E3 ubiquitin ligase, leads to the conjugation of ubiquitin in various mitochondrial substrates, such as mitofusin1 and 2 (MFN1/2), mediating their proteasomal degradation.[qv: 21] We found that BRCA1 deficiency had no effect on the ubiquitin level of mitochondria or the degradation of MFN1 and MFN2 after CCCP treatment (Figure S2C,D, Supporting Information). These results demonstrate that BRCA1 regulates stress‐induced mitophagy independent of PINK1/Parkin activation.

**Figure 2:**
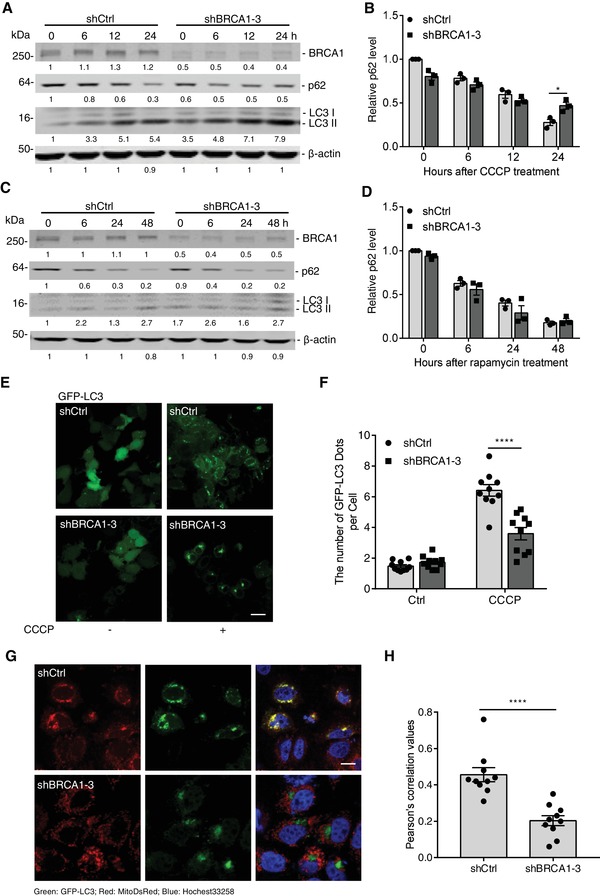
Loss of BRCA1 impairs autophagosome formation during mitophagy. A) Immunoblot analysis of BRCA1, p62, and LC3 in shCtrl and shBRCA1 Hela‐mCherryParkin under CCCP treatment. B) Quantification of p62 level in (A), which normalized by β‐actin level (*n* = 3 per group). C) Immunoblot analysis of BRCA1, p62, and LC3 in shCtrl and shBRCA1 Hela‐mCherryParkin under rapamycin (5 × 10^−6^ m) treatment. D) Quantification of the p62 level in (C), normalized by the β‐actin level (*n* = 3 per group). E) Representative fluorescent images of the puncta formation of GFP‐LC3 in shCtrl and shBRCA1 Hela‐mCherryParkin infected with GFP‐LC3 retrovirus, with or without CCCP treatment, for 6 h. Scale bar, 20 µm. F) Quantification of GFP‐LC3 puncta in (E) (ten fields counted per group). G) Representative fluorescent images of the colocalization of GFP‐LC3 with mitochondria (labeled by MitoDsRed) in Hela‐HA‐Parkin infected with retrovirus‐GFP‐LC3 and lentivirus‐MitoDsRed while under CCCP treatment. Hochest33258, DNA‐binding dye. Scale bar, 10 µm. H) Pearson's coefficient is shown as the quantification of GFP‐LC3 puncta colocalized with mitochondria per cell in (G) (ten fields counted per group). Data represent the mean ± SEM and are representative of three independent experiments. Significant differences were determined by two‐way ANOVA with Tukey's multiple comparison test (B, D, and F) or an unpaired two‐tailed *t*‐test (H) *p < 0.05, ****p < 0.0001.

Mitophagy occurs through general autophagic machinery, so we sought to determine whether BRCA1 might regulate mitophagy by mediating general autophagic activity. As shown in **Figure** **2**A,B, BRCA1 KD inhibited p62 degradation under CCCP treatment, but caused greater accumulation of the lipidated LC3 form (LC3‐II), indicating that BRCA1 deficiency blocks CCCP‐induced autophagic flux. Unexpectedly, the general autophagy activity induced by rapamycin or hank's balanced salt solution (HBSS) was not altered by loss of BRCA1, as indicated by lipidation of LC3 and degradation of p62 (Figure 2C,D and Figure S3A, Supporting Information). This suggests that BRCA1 regulates mitophagy irrespective of general autophagic pathway.

It was found that the recruitment of autophagosomes to mitochondria facilitates subsequent selective autophagy in response to mitochondrial stress.[qv: 11] To address why the loss of BRCA1 suppresses autophagic flux, we monitored autophagosome formation by quantification of GFP‐LC3 punctuation (a marker of autophagosome) in shCtrl and shBRCA1 cells, expressing GFP‐LC3 after CCCP treatment. GFP‐LC3 puncta were dramatically decreased in shBRCA1 cells compared to shCtrl cells, and the colocalization of LC3‐GFP and mitochondrion was also reduced in BRCA1 KD cells (Figure 2E–H). In addition, lysosomes, marked with LAMP1‐red fluorescent protein (RFP), were colocalized more significantly with shCtrl mitochondria compared to shBRCA1 mitochondria (Figure S3B,C, Supporting Information). This finding indicates that BRCA1 deficiency inhibits stress‐induced mitophagy through blocking autophagosome formation.

### 2.4 BRCA1 Negatively Regulates Mitochondrial Fusion

**Figure 3:**
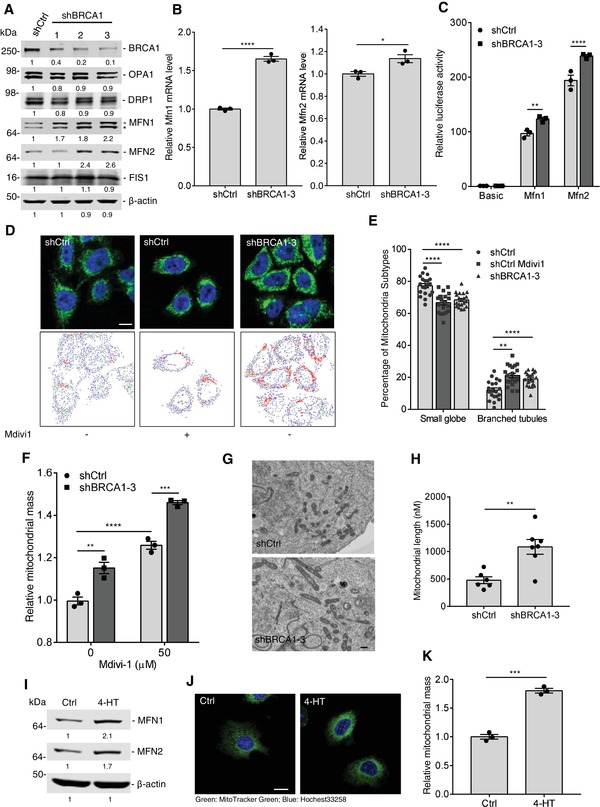
Loss of BRCA1 increases MFN1/2 expression and enhances mitochondrial fusion. A) Immunoblot analysis of BRCA1, MFN1, MFN2, OPA1, DRP1, and FIS1 in shCtrl and shBRCA1 MDA‐MB‐231 cells with different BRCA1 shRNA. Asterisk (*) indicates MFN2 band. B) The mRNA levels of Mfn1 and Mfn2 in shCtrl and shBRCA1 MDA‐MB‐231 cells (*n* = 3 per group). C) The promoter activities of *Mfn1* and *Mfn2* in shCtrl and shBRCA1 MDA‐MB‐231 cells by measuring luciferase activity. Basic, pGL3‐Basic; Mfn1/Mfn2, pGL3‐Basic driven by *Mfn1* or *Mfn2* promoter (*n* = 3 per group). D) Mitochondrial morphology of shCtrl and shBRCA1 MDA‐MB‐231 cells. shCtrl cells were treated by Mdivi1 (50 × 10^−6^ m) for 24 h. Upper panel: Representative fluorescent images of mitochondrial morphology stained by MitoTracker Green, Scale bar, 10 µm. Lower panel: Micro‐P analytic images of mitochondrial morphology, Micro‐P algorithm classified mitochondria into small globe mitochondrion (blue) and branched mitochondrion (red). E) Percentage of small globe subtype and branched subtype classified by Micro‐P (20 cells counted per group). F) The mitochondrial mass in shCtrl and shBRCA1 MDA‐MB‐231 cells with or without Mdivi1 treatment (*n* = 3 per group). G) Electron microscopy analysis of mitochondrial morphology in shCtrl and shBRCA1 MDA‐MB‐231 cells. Scale bar, 500 nm. H) Quantification for mitochondrial lengths by ImageJ (six to seven fields counted per group). I) Immunoblot analysis of MFN1 and MFN2 in *Brca1^flox/flox^*; *Tam‐Cre* MEFs with or without 4‐HT (5 × 10^−6^ m) treatment. J) Mitochondrial morphology of *Brca1^flox/flox^*; *Tam‐Cre* MEFs with or without 4‐HT treatment, stained by MitoTracker Green. Scale bar, 10 µm. K) The mitochondrial mass in *Brca1^flox/flox^*; *Tam‐Cre* MEFs with or without 4‐HT treatment (*n* = 3 per group). Data represent the mean ± SEM and are representative of three independent experiments. Significant differences were determined by an unpaired two‐tailed *t*‐test (B, H, and K) or ANOVA with Tukey's multiple comparison test (C, E, and F). *p < 0.05, **p < 0.01, ***p < 0.001, ****p < 0.0001.

Mitochondria are dynamic organelles, which continually change shape through the combined action of fission, fusion, and movement along cytoskeletal tracks.[qv: 22] Our results indicate that expression of MFN1/2 was elevated in shBRCA1 cells compared to shCtrl cells in the absence of CCCP treatment (Figure S2D, Supporting Information), suggesting that BRCA1 might regulate mitochondrial dynamics in breast cancer cells. To corroborate our hypothesis, the expression of mitochondrial dynamics‐related proteins was measured in MDA‐MB‐231 cells treated with shCtrl or shBRCA1. The results indicate that expression of MFN1/2 increases in shBRCA1 cells at both the protein and RNA levels through enhancing the promoter activities of *Mfn1* and *Mfn2* (**Figure** **3**A–C), but other dynamic proteins, such as optic atrophy type 1 (OPA1), dynamin‐related protein 1 (DRP1), and mitochondrial fission protein 1 (FIS1), showed no significant difference (Figure 3A). These results suggest that BRCA1 KD enhances mitochondrial fusion by promoting MFN1/2 expression. We then examined if BRCA1 had an effect on the morphology of mitochondria by using MitoTracker Green FM. As shown in Figure 3D, shBRCA1 cells display a more condensed mitochondrial network, similar to shCtrl cells treated with mitochondrial division inhibitor 1 (Mdivi‐1). We further quantified BRCA1‐mediated mitochondrial‐morphological changes using an automated system for the quantification and classification of mitochondrial morphology, Micro‐P.[qv: 23] According to Micro‐P analysis, shBRCA1 cells displayed a significantly higher percentage of the branched tubule type of mitochondrion with a decrease in the small globe type (Figure 3E). Because mitochondrial fusion increases mitochondrial mass, we investigated this in shCtrl and BRCA1 KD cells with or without Mdivi‐1 treatment through flow cytometry. The results showed that BRCA1 KD significantly increased mitochondrial mass compared to shCtrl cells; and similar increase was also observed in shCtrl cells after Mdivi‐1 treatment (Figure 3F). In addition, electron microscopy revealed that more elongated mitochondria in shBRCA1 cells than in shCtrl cells (Figure 3G,H).

To further confirm the effect of BRCA1 on mitochondrial fusion, we examined MFN1/2 expression and mitochondrial morphology in *Brca1^flox/flox^* MEFs with *Tam‐Cre* along with 4‐HTtreatment. Likewise, loss of *Brca1* enhanced mitochondrial fusion through increased MFN1/2 expression (Figure 3I–K and Figure S4A, Supporting Information). On the contrary, the restoration of BRCA1 expression in the BRCA1 mutant HCC1937 cell line decreased MFN1/2 expression and changed mitochondrial morphology from tubular networks to fragmented puncta (Figure S4B–D, Supporting Information). In addition, we found that *BRCA1* mutation increased MFN1/2 expression in breast cancer patient‐derived xenograft (PDX) models (Figure S4E,F, Supporting Information). Excessive mitochondrial fusion could block segregation of damaged organelles from the healthy mitochondrial network and cause more mitochondrial damage. Therefore, these combined data indicate that BRCA1 could regulate mitochondrial fusion and maintain a healthy mitochondrial network.

### 2.5 BRCA1 Is Required for Stress‐Induced Mitochondrial Fission through the Mediation of AMPK Activation

**Figure 4:**
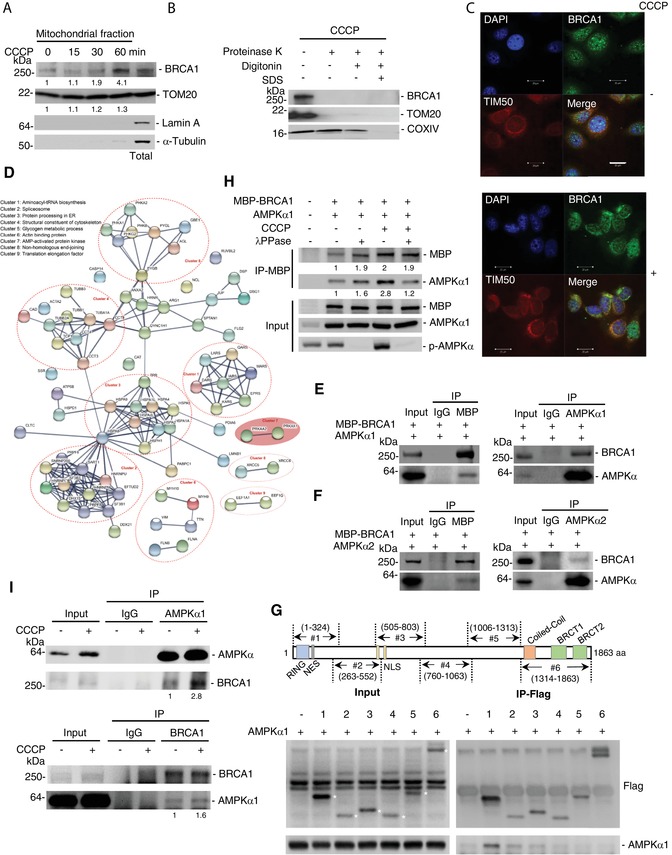
BRCA1 interacts with AMPKα on the mitochondria. A) The localization of BRCA1 on mitochondria in MCF7 cells under CCCP treatment at the indicated time points. Total, total cell protein. α‐Tubulin and Lamin A are load control for cytoplasmic and nuclear protein, respectively. B) BRCA1 localized on outer‐membrane of mitochondria under CCCP treatment. MCF cells were treated with CCCP for 1 h. TOM20 and COXIV are indicators for mitochondrial outer‐ and inner‐membrane protein, respectively. C) Immunofluorescent images of BRCA1 in MCF7 cells with or without CCCP treatment. Mitochondria labeled by immunostaining for TIM50. Scale bar, 20 µm. D) Network of proteins interacting with BRCA1 analyzed by STRING. Cluster 7 was highlighted. E,F) CoIP analysis of the interaction of BRCA1 and AMPKα1/2 in 293T cells transfected with MBP‐BRCA1 and AMPKα1/2‐expressing vectors. G) BRCA1 interacts with AMPKα through its RING domain. Upper panel: Six overlapping fragments that cover the entire BRCA1 protein. Lower panel: Analysis of the interaction between AMPKα1 and BRCA1 fragments. Asterisks indicate the positions of BRCA1 fragments. H) Phosphorylation of AMPKα is required for its interaction with BRCA1 under CCCP treatment. 293T cells were treated or not with CCCP after transfected with MBP‐BRCA1 and AMPKα1‐expressing vectors. Cell lysates were treated or not with λ‐phosphatase (λPPase) for 30 min before immunoprecipitation. I) CoIP analysis of interaction of endogenous BRCA1 and AMPKα1 on mitochondria of MCF7 cells. Mitochondrial protein exacted from MCF7 cells with or without CCCP treatment, with CoIP analysis performed. Upper panel: Immunoprecipitation performed by AMPKα1 antibody. Lower panel: Immunoprecipitation performed by BRCA1 antibody. Data are representative of at least two independent experiments.

For the initiation of mitophagy, the mitochondrial network must be divided into smaller mitochondria through fission[qv: 24] and degradation of mitofusin induced by PINK1/Parkin could be a means of preventing mitochondrial fusion.[qv: 21] Our finding that BRCA1 deficiency could promote mitochondrial fusion by increasing the level of MFN1/2 (Figure 3), which suggests that excessive fusion may lead to the failure of autophagosome formation and a mitophagy defect in *BRCA1* deficient cells. However, we also found that mitochondrial damage with CCCP promptly induced MFN1/2 degradation in both Ctrl and BRCA1 KD cells (Figure S2D, Supporting Information), indicating how BRCA1 regulates stress‐induced mitophagy is not clear. To address the effect of BRCA1 on stress‐induced mitophagy, we examined mitochondrial localization of BRCA1 under mitochondrial stress. We found that BRCA1 translocated onto mitochondria after CCCP treatment (**Figure** **4**A) and was removed by treatment with proteinase K (Figure 4B), demonstrating that BRCA1 is sensitive to proteinase K treatment and localizes on the outer membrane of mitochondria: this is similar to other outer membrane proteins such as TOM20. A subcellular distribution pattern of BRCA1 by immunofluorescence confirmed CCCP‐induced localization of BRCA1 to mitochondria (Figure 4C). To avoid nonspecific issue that might occur with antibody, we also generated Flag‐tagged BRCA1 MEFs for detecting BRCA1 localization under CCCP treatment (Figure S5A, Supporting Information), and the data confirmed that CCCP caused mitochondrial translocation of BRCA1 in MEFs, as detected by the Flag antibody (Figure S5B,C, Supporting Information).

To identify the potential partners that interact with BRCA1 during mitophagy, maltose binding protein (MBP)‐tagged BRCA1 was overexpressed in 293T cells and immunoprecipitated by the MBP antibody. We pulled down 74 proteins that contained more than four unique identified peptides, indicating that these proteins could interact with BRCA1 (Table S1, Supporting Information). We then generated the protein–protein interaction network based on molecular functions by STRING. The network shows the five big interaction clusters involved in tRNA biosynthesis, pre‐mRNA splicing, protein processing, cytoskeleton, and glycogen metabolism, but these clusters have little to do with mitophagy (Figure 4D). However, cluster 7, containing PRKAA1 and PRKAA2, caught our attention. The cluster is involved in two catalytic subunits of AMP‐activated protein kinase (AMPK), AMPKα1 and AMPKα2 (Figure 4D). AMPK is a highly conserved metabolic sensor that plays a strong role in regulating cell growth, metabolism, and autophagy.[qv: 25] It is suggested that BRCA1 might regulate stress‐induced mitophagy dependent on AMPK signaling activation. To substantiate our hypothesis, we first validated the interaction of BRCA1 with AMPKα1 and AMPKα2. The MBP‐BRCA1 construct with corresponding candidate‐expressing constructs were transfected into 293T cells and the interaction was checked by Co‐immunoprecipitation (CoIP) and Western blotting. The results showed that MBP‐BRCA1 could interact reciprocally with AMPKα1 and AMPKα2 (Figure 4E,F).

BRCA1 is a multidomain protein that contains N‐terminal RING domain, Coiled‐Coil domain, BRCA1 C‐terminal (BRCT) repeats, nuclear export sequence (NES), and two nuclear localization sequences.[qv: 26,27] We investigated the ability of AMPKα1 to interact with six Flag‐tagged BRCA1 fragments, and found that fragment #1, containing the RING domain and NES, interacted with AMPKα1 (Figure 4G). Further results indicated that CCCP‐induced mitochondrial damage enhanced the interaction of BRCA1 with AMPKα1, which is dependent on phosphorylation of AMPKα (Figure 4H). Furthermore, to validate the interaction in the mitochondria, we extracted mitochondrial proteins from MCF7 cells, with or without CCCP treatment, and examined the connection between endogenous BRCA1 with AMPKα1. As shown in Figure 4I, BRCA1 and AMPKα1 interacted reciprocally, which was enhanced by CCCP treatment. These results suggest that BRCA1 could play a role in AMPK signaling pathway.

**Figure 5:**
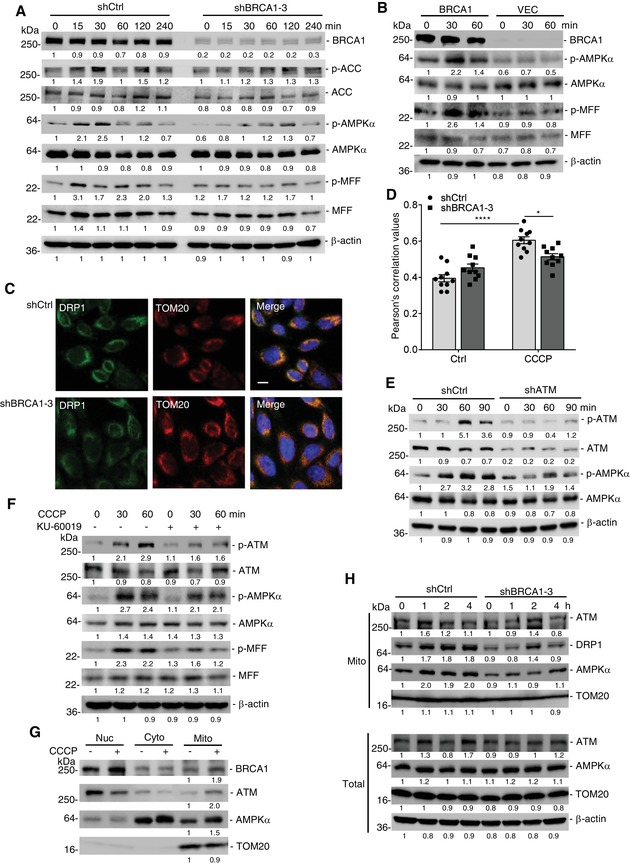
Loss of BRCA1 impairs mitochondrial localization of DRP1 via blocking activation of ATM‐AMPK‐MFF pathway. A) BRCA1 KD suppressed CCCP‐induced AMPK activation in 293T cells. Cells were treated by CCCP (10 × 10^−6^ m) for indicated time points. B) Ectopic expression of BRCA1 restored CCCP‐induced AMPK activation in BRCA1 mutant HCC1937 cells. The cells stably transfected with vector (VEC) or BRCA1‐expressing vector (BRCA1) and treated by CCCP for indicated time points. C) Representative fluorescent images of the localization of DRP1 on mitochondria in shCtrl and shBRCA1 Hela cells after CCCP treatment for 1 h. Mitochondria were labeled by staining with TOM20 antibody. DAPI, DNA‐binding dye. Scale bar, 10 µm. D) Pearson's coefficient is shown as the quantification of DRP1 colocalized with mitochondria per cell in (C) (ten fields counted per group). E,F) Inhibition of ATM suppressed CCCP‐induced AMPK activation in 293T cells. E) shCtrl and shATM 293T cells were treated by CCCP for indicated time points; F) 293T cells were pretreated by ATM inhibitor KU‐60019 (5 × 10^−6^ m) for 30 min, then treated by CCCP for indicated time points. G) Immunoblot analysis of cellular localization of BRCA1, ATM, and AMPKα in MCF7 cells before and after CCCP treatment for 1 h. Nuc, nuclear protein; Cyto, cytoplasmic protein; Mito, mitochondrial protein. H) Immunoblot analysis of ATM, AMPKα, and DRP1 levels on mitochondria in shCtrl and shBRCA1 MCF7 cells under CCCP treatment. Total, total cell protein. Data represent the mean ± SEM and are representative of at least two independent experiments. Significant differences were determined by two‐way ANOVA with Tukey's multiple comparison test. *p < 0.05, ****p < 0.0001.

A recent study indicated that AMPK could regulate mitochondrial fission and subsequent mitophagy via inducing the phosphorylation of MFF (mitochondrial fission factor).[qv: 28] Therefore, we investigated whether BRCA1 regulated AMPK activation and mitochondrial fission with CCCP treatment. We found that BRCA1 KD could impair AMPK activation, as reflected by decreased phosphorylation of AMPKα and its known downstream targets, such as Acetyl‐CoA Carboxylase (ACC) and MFF post‐CCCP treatment (**Figure** **5**A). BRCA1 deficiency also impaired oligomycin‐induced AMPK activation (Figure S5D, Supporting Information). Conversely, ectopic expression of BRCA1 could restore phosphorylation of AMPK and MFF and sensitize additional phosphorylation with CCCP treatment (Figure 5B). However, BRCA1 had no effect on AMPK activation under AMPK inducer 5‐aminoimidazole‐4‐carboxamide riboside (AICAR) treatment (Figure S5E, Supporting Information), suggesting that BRCA1 is essential for AMPK activation with mitochondrial damage. Because activated AMPK phosphorylation of MFF is required for mitochondrial localization of DRP1 and mitochondrial fission,[qv: 28] we investigated whether BRCA1 affected DRP1 localization after CCCP treatment. As shown in Figure 5C,D, BRCA1 KD inhibited CCCP‐induced recruitment of DRP1 to mitochondria. Consequently, it blocked DRP1‐mediated segregation of damaged mitochondria, reflected by more compact mitochondria in shBRCA1 cells on the CCCP treatment, compared with shCtrl cells (Figure S5F,G and Movie S1,2, Supporting Information). Mitochondrial fission generates smaller mitochondria, which can easily be sequestered by autophagosomal membrane; therefore, DRP1 deficiency could impair mitophagy under CCCP treatment, reflected by the degradation of mitochondrial proteins such as HSP60 and COXII (Figure S5H, Supporting Information). These combined data suggest that loss of BRCA1 inhibits AMPK‐mediated MFF phosphorylation and DRP1 mitochondrial translocation and blocks stress‐induced mitophagy due to defective mitochondrial fission.

### 2.6 BRCA1 Is Required for Stress‐Induced AMPK Activation through Mediating the Localization of ATM‐AMPKα in the Mitochondrion

It is well known that liver kinase B1 (LKB1) and calcium/calmodulin‐dependent protein kinase kinase‐β (CaMKKβ), two major upstream kinases, activate AMPK by phosphorylating Thr172 in the activation loop of the catalytic α‐subunit.[qv: 25] To investigate the upstream kinase of CCCP‐induced AMPK activation, we examined whether LKB1 or CaMKKβ were required for CCCP‐induced AMPKα and MFF phosphorylation. The results showed that LKB1 or CaMKKβ KD had no effect on the phosphorylation of AMPKα and MFF under CCCP treatment (Figure S6A,B, Supporting Information). Meanwhile, AMPK activation induced by CCCP was unaffected in Hela cells, which lacks LKB1; however, it was blocked by shBRCA1 (Figure S6C, Supporting Information). These results indicate that AMPK activation induced by CCCP is BRCA1‐dependent but was not LKB1‐ or CaMKKβ‐dependent.

It was shown that ATM, an upstream kinase, could phosphorylate AMPKα both in vivo and in vitro.[qv: 29] We then examined whether ATM was involved in CCCP‐induced AMPK activation. The data indicated that CCCP treatment induced phosphorylation of ATM and AMPK, and inhibition of ATM activity by ATM KD or ATM inhibitor (KU‐60019) led to impairment of AMPK activation (Figure 5E,F), which demonstrated that ATM is required for CCCP‐induced AMPK activation. It is known that BRCA1 facilitates the ability of ATM to phosphorylate downstream substrates under DNA damage treatment;[qv: 30] as such, we hypothesized that BRCA1 might be required for ATM‐mediated AMPK activation. To corroborate this, we first investigated the localization of BRCA1, ATM, and AMPKα before and after CCCP treatment. As shown in Figure 5G, CCCP treatment could not only cause translocation of BRCA1 onto mitochondria but also increased levels of ATM and AMPKα on the mitochondrion. We found that BRCA1 deficiency impaired mitochondrial translocation of ATM and AMPKα after CCCP treatment for 0.5 h (Figure 5H), so the level of DRP1 on the mitochondria decreased in shBRCA1 cells when compared to shCtrl cells; this confirms localization of DRP1 as measured by immunofluorescence (Figure 5C,D). These data indicate that the loss of BRCA1 inhibits ATM‐mediated AMPK activation through blocking mitochondrial localization of ATM and AMPKα.

### 2.7 Loss of BRCA1 Leads to Inflammasome Activation

**Figure 6:**
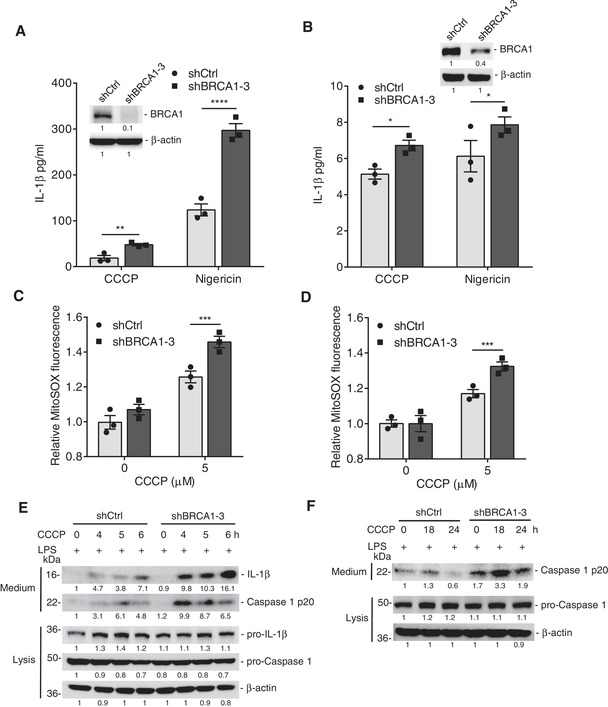
BRCA1 deficiency promotes inflammasome activation. A) Release of IL‐1β from LPS‐primed THP‐1 cells and B) MCF10A cells stimulated with CCCP or Nigericin, as measured by ELISA (*n* = 3 per group). C) Measurement of mitochondrial ROS in THP‐1 cells and D) MCF10A cells (*n* = 3 per group). E) Immunoblot analysis of pro‐caspase 1 and pro‐IL‐1β processing in LPS‐primed THP‐1 cells and F) MCF10A cells stimulated as indicated. Data represent the mean ± SEM and are representative of three independent experiments. Significant differences were determined by two‐way ANOVA with Sidak's multiple comparison test (A and B) or with Tukey's multiple comparison test (C and D). *p < 0.05, **p < 0.01, ***p < 0.001, ****p < 0.0001.

It has been reported that defective mitophagy leads to the accumulation of damaged mitochondria and excessive ROS, which then activates the NLRP3 inflammasome.[qv: 12] We found that damaged mitochondria and ROS accumulated in BRCA1‐deficient cells (Figure 1G–I). Pathway enrichment and GSEA analysis indicated that the inflammatory response significantly increased in *Brca1* mutant MG (Figure 1A and Figure S7, Supporting Information). These results suggest that loss of BRCA1 might lead to activation of inflammasome. To confirm this, we first used a human monocytic cell line (THP‐1 cells), which represents the most commonly used cells to assess the inflammasome activation. We found that nigericin, an inflammasome inducer, could dramatically promote activation in BRCA1 KD cells, compared to control cells (**Figure** **6**A). Meanwhile, CCCP‐induced mitochondrial stress enhanced ROS production and generated more cleaved caspase 1 and mature interleukin‐1β (IL‐1β) in BRCA1 KD cells (Figure 6C,E). To establish the effect of BRCA1 on inflammasome activation in mammary cells, we analyzed IL‐1β secretion and caspase 1 activation in MCF10A cells with detectable inflammasome‐associated components, compared to several breast cancer cell lines (Figure S8, Supporting Information). The results showed that BRCA1 KD led to more ROS production and inflammasome activation in MCF10A cells (Figure 6B,D,F), which indicates that BRCA1 deficiency can trigger inflammasome activation.

### 2.8 Blocking Inflammasome Activity Ameliorates *Brca1* Mutant Mammary Tumor Recurrence and Metastasis

**Figure 7:**
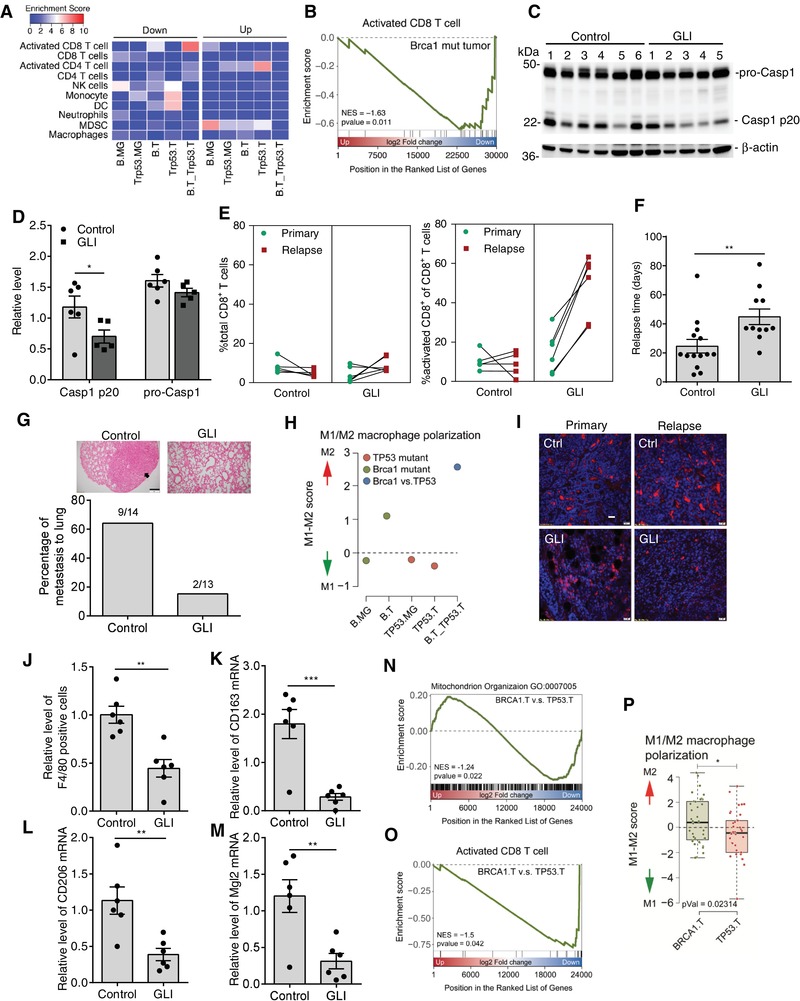
Excessive inflammasome activation promotes mammary tumor recurrence and metastasis. A) Immunocytes profile analysis in *Brca1* mutant MG (B.MG), *Brca1* mutant tumor (B.T), *Trp53* mutant MG (TP53.MG), and *Trp53* mutant tumor (Trp53.T) after normalization with WT MG or in B.T after compared with Trp53.T (B.T_Trp53.T) according to the RNA‐Seq. B) GSEA plot of enrichment in “activated CD8 T cell” gene set significantly downregulated in B.T versus Trp53.T. C) Immunoblot analysis of procaspase 1 (proCasp1) processing in recurrent tumors with or without GLI treatment. D) Quantification of proCasp1 and Casp1 p20 level in (C), normalized by β‐actin level (*n*_control_ = 6, *n*_GLI_ = 5). E) CD8^+^ T cells and activated CD8^+^ T cells in primary and recurrent tumors analyzed by flow cytometry with or without GLI treatment (*n*_control_ = 5, *n*_GLI_ = 6). F) Relapse time of tumors after removing primary mammary tumors from *Brca1^flox/flox^;MMTV‐Cre* mice with or without GLI treatment (*n*_control_ = 14, *n*_GLI_ = 13, two mice in GLI group show no relapse). G) Representative images of lung with hemotoxylin and eosin (H&E) staining sections from *Brca1^flox/flox^; MMTV‐Cre* mice after primary mammary tumor removal with or without GLI treatment. Arrow indicates metastatic tumor on the lung. Scale bar, 200 µm. H) M1/M2 macrophage polarization analysis in indicated groups. I) Representative fluorescent images of primary and recurrent tumor sections with immunostaining for F4/80. Scale bar, 20 µm. J) Quantification of F4/80 positive macrophages in recurrent tumors, relative to those in primary tumors with or without GLI treatment (*n*_control_ = 6, *n*_GLI_ = 6). K–M) The mRNA level of M2 macrophage markers in recurrent tumors after normalization by those in primary tumors with or without GLI treatment, measured by real‐time PCR (*n*_control_ = 6, *n*_GLI_ = 6). N) GSEA plot of enrichment in “mitochondrion organization” gene set significantly downregulated in breast cancers carrying *BRCA1* mutation (BRCA1.T) versus cancers carrying *TP53* R175H mutation (TP53.T) according to the METABRIC database. O) GSEA plot of enrichment in “activated CD8 T cell” gene set downregulated in BRCA1.T versus TP53.T. P) M1/M2 macrophage polarization analysis in BRCA1.T compared with that of TP53.T. Data represent the mean ± SEM. Significant differences were determined by two‐way ANOVA with Sidak's multiple comparison test (D) or an unpaired two‐tailed *t*‐test (F, J, K, L, M, and P). *p < 0.05, **p < 0.01, ***p < 0.001.

IL‐1β promotes tumor progression and counteracts immunosurveillance by recruiting myeloid cells, such as myeloid‐derived suppressor cells (MDSCs) and tumor‐associated macrophages (TAMs).[qv: 31,32] This suggests that BRCA1 mutation could establish a tumor‐associated microenvironment for cancer progression via inflammasome activation. To verify the hypothesis, we first investigated immunocytes' profiles in the *Brca1* mutant MG and mammary tumors from *Brca1^flox/flox^;MMTV‐Cre* mice, which frequently develop a single mammary tumor.[qv: 33,34] We also analyzed the immunocytes' profiles in *Trp53* mutant MG and mammary tumors from *Trp53^flox/flox^;MMTV‐Cre* mice, since *TP53* mutations are the second most frequent genetic alteration in breast cancer, observed in 23% of them, according to the catalog of somatic mutations in cancer database.[qv: 35] Although total T cells, including CD4^+^ and CD8^+^ T cells, have no significant difference in these groups, activated CD8^+^ T cells significantly downregulate in *Brca1*mutant tumor compared to WT MG or *Trp53* mutant tumor (**Figure** **7**A,B). The results indicate that inflammasome activation triggered by BRCA1 deficiency leads to impairment of CD8^+^ T cell activity. To confirm this, we investigated whether inhibition of inflammasome activity could reactivate CD8^+^ T cells and alleviate *Brca1* mutant tumor progression. As shown in Figure S9A in the Supporting Information, we surgically removed primary mammary tumors and observed tumor relapse and metastasis with or without inflammasome inhibitor (glibenclamide, GLI) treatment. In according with our expectations, GLI treatment inhibited inflammasome activity in recurrent tumors as measured by cleaved caspase 1 level (Figure 7C,D), and the treatment dramatically increased the percentage of activated CD8^+^ T cells in the total number of CD8^+^ T cells, which was not affected by the inhibitor (Figure 7E and Figure S9B, Supporting Information). We found that the treatment delayed tumor recurrence (Figure 7F) and blocked lung metastasis of the *Brca1* mutant tumor (15.3%) compared to no treatment group (64.2%) (Figure 7G and Figure S9C, Supporting Information). Two mice injected with GLI had no relapse for up to six months. Our results support that inflammasome activation in *Brca1*mutant tumor impairs CD8^+^ T cell activation and promotes tumor progression.

As mentioned above, MDSCs and TAMs are key components of the tumor‐associated microenvironment that promotes tumor progression and metastasis.[qv: 31,32] MDSCs rather than other myeloid cells, such as neutrophils and monocytes/macrophages, significantly increased in *Brca1* mutant MG (Figure 7A), but not in *Brca1* mutant tumor (Figure S9D, Supporting Information). This suggests that MDSCs may play a role in the initiation of *Brca1* mutant tumor, not in tumor progression. We found that although the level of macrophages in *Brca1* mutant tumors was not different from that in *Trp53* mutant tumors (Figure S9E, Supporting Information), *Brca1* mutant tumors displayed unbalanced M1/M2 macrophage polarization compared to other groups (Figure 7H). It is well known that most TAMs are considered to have an M2 phenotype, while playing a crucial role in building an immunosuppressive environment that blocks T cell activation.[qv: 36] The results show that more polarized M2 macrophages were recruited into *Brca1* mutant tumor compared with those in WT MG and *Trp53* mutant tumors (Figure 7H). Moreover, we found that GLI treatment reduced the percentage of macrophages in relapse tumors, compared with those in primary tumors, which were analyzed by macrophage marker F4/80 staining (Figure 7I,J). The M2 polarization of TAM could be demonstrated by analyzing their transcriptional profiling.[qv: 36] Therefore, we measured the mRNA levels of M2 macrophage markers such as CD163, CD206, and Mgl2 in recurrent tumors and in primary tumors with or without GLI treatment. This shows that the levels of these markers significantly decrease in recurrent tumors treated with GLI after normalizing those levels in primary tumors (Figure 7K–M). These data suggest that macrophages infiltrating tumor tissues were driven by *Brca1* mutant tumor‐derived cytokines to acquire a polarized M2 phenotype, which impairs CD8^+^ T cells activation.

To verify this phenomenon in clinical samples, we analyzed immunocyte profiles in breast cancers developed in patients carrying the pathogenic *BRCA1* mutations in the Molecular Taxonomy of Breast Cancer International Consortium (METABRIC) database.[qv: 37] It was reported that nearly all luminal breast cancers carry point mutations in *TP53*,[qv: 38] so we used breast cancer patients with a *TP53* R175 mutation, a hotspot mutation as a control group. This demonstrates that mitochondrial function is impaired in *BRCA1* mutant tumors, while enriched genes correspond to mitochondrial organization (Figure 7N), and *BRCA1* mutant tumor accumulates more M2‐polarized macrophages, as well as containing less activated CD8^+^ T cells than the *TP53* mutant tumors (Figure 7O,P). These observations show that *BRCA1* mutation can cause abnormal M2 macrophage recruitment to suppress CD8^+^ T cell activation in clinical samples.

The data suggest that blocking excessive inflammasome activity in *Brca1* mutant tumor can postpone tumor recurrence and inhibit tumor metastasis through impaired the recruitment of TAMs.

## 3 Discussion

BRCA1 is a well‐known tumor suppressor, having been linked to a range of cellular processes such as DNA repair, transcriptional regulation, chromatin remodeling, and cell cycle checkpoints.[qv: 5,6] While these BRCA1 functions are largely attributed to nuclear localization, the role of BRCA1 in the cytoplasm remains elusive. Although a recent study has indicated that multiple Fanconi anemia pathway genes including BRCA1 are required for mitophagy,[qv: 39] its underlying mechanism remains poorly defined, and the relationship of defective mitophagy with BRCA1‐associated breast cancer development is unknown. In this study, we reveal that BRCA1 is located at the mitochondrial membrane at a low level but is significantly enhanced under stress conditions. Moreover, BRCA1 plays an important role in maintaining mitophagy through involvement in mitochondrial fission and fusion. BRCA1 deficiency blocks mitophagy, leading to the accumulation of damaged mitochondria and inflammation which is involved in tumorigenesis and cancer metastasis.

The mitochondrion is a highly complex organelle, which dynamically changes its morphology and network through continuously undergoing two opposite processes: fusion and fission.[qv: 10] It has been reported that deletion of BRCA1 leads to enlarged mitochondria in skeletal muscle,[qv: 16,40] indicating that BRCA1 deficiency causes dysfunctional mitochondrial dynamics, yet the underlying mechanism has not been illustrated. Our data demonstrated BRCA1 negatively regulates mitochondrial fusion through inhibiting expression of MFN1/2 at the transcriptional level. Both MFN1 and MFN2 are GTPases localized in the outer membrane of the mitochondrion. With some other proteins, such as OPA1, whose expression is not affected by BRCA1, these proteins take part in the fusion process.[qv: 22] Loss of BRCA1 results in a higher level of MFN1/2 proteins, accelerating mitochondrial fusion, which can block the separation of damaged mitochondria from healthy ones and negatively affect mitophagy.

It has been reported that BRCA1 interacts with ACC, one of the downstream targets of AMPK, by pulling down BRCT residues of BRCA1,[qv: 41] and BRCA1 stabilizes phosphorylation of ACC to affect lipid synthesis via this interaction.[qv: 42] In this study, full length of BRCA1 was pulled down to screen for interacting proteins. It is determined that AMPKα1/2 interacts with BRCA1 via the RING domain, and BRCA1 positively regulates AMPK signaling activation under mitochondrial damage, not AICAR treatment, demonstrating that a novel function of BRCA1 on AMPK signaling.

Phosphorylated BRCA1[qv: 43] or a number of isoforms of BRCA1[qv: 44] are located inside the mitochondria. To elucidate the molecular mechanism between BRCA1 and CCCP‐induced AMPK activation, we deciphered localization of BRCA1 on mitochondria with or without mitochondrial damage. Our data indicated that BRCA1 is located on the outer membrane of mitochondria at low levels under normal conditions, as it can be degraded with proteinase K treatment similar to other outer membrane proteins, as mitochondrial stress promotes more BRCA1 translocation to mitochondria. Although BRCA1 has no mitochondrial targeting signal, it is known that its main binding partner, BARD1, can translocate on mitochondria that depend on BRCT domain.[qv: 45,46]It suggests that mitochondrial translocation of BRCA1 may be regulated by its own BRCT domain or interaction with BRAD1, which will be addressed in future study.

Furthermore, the increased BRCA1 on the outer membrane of mitochondria is correlated with the activation of AMPK and its downstream proteins, while the recruitment of DRP1 to the mitochondrial outer membrane plays a central role in triggering mitochondrial fission. We found BRCA1 is required for the translocation of ATM onto mitochondria to promote activation of AMPK under stress condition, which provides a link in the recruitment of DRP1 to mitochondria with BRCA1. These findings demonstrate that BRCA1‐ATM‐AMPK‐DRP1 signaling together to regulate mitochondrial fission.

Thus, BRCA1 deficiency affects mitochondrial fusion through increasing the transcription of MFN1/2 and affects fission through impairing stress‐induced AMPK activation and DRP1‐mediated mitochondrial fission. These effects can result in elongated mitochondria and inhibit the dynamic balance of mitochondria in *BRCA1* mutant cells, leading to the failure of mitophagy: the initiation of mitophagy requires the mitochondrial network to be divided into smaller mitochondria through fission.[qv: 24] These data uncover an important function of BRCA1 at the mitochondrial outer membrane that regulates mitophagy.

It has been shown that insufficient clearance of dysfunctional mitochondria, which can result from defective mitophagy, has been implicated in a series of pathophysiological conditions, including inflammasome activation, genotoxic stress, and promotion of tumorigenesis.[qv: 12,13] Another important finding is that loss of BRCA1 triggers inflammasome activation which then promotes *Brca1* mutant tumor relapse and metastasis. In this study, upregulated inflammasome activation enriches TAMs' recruitment to suppress CD8^+^ T cell activation in *Brca1* mutant mammary tumors; thus, inhibition of inflammasome activation could postpone tumor relapse and block lung metastasis after surgically removing primary cancers. It is known that metastatic dormancy complicates the treatment of breast cancer, as surgical excision of primary tumors results in the acceleration of metastatic tumors.[qv: 47] Our findings suggest that the interference of inflammasome activation could protect *BRCA1* mutant breast cancer patients from metastatic recurrence after resecting primary tumors. These results also provide more evidence to support recent findings that anti‐inflammatory treatment reduces the incidence of early metastatic relapse in breast cancer patients and decreases tumor growth in mice models for dormancy.[qv: 47,48] Interestingly, our result shows that *Brca1* mutant‐associated tumor microenvironment is different from that of *Trp53* mutant‐associated type. Activated CD8^+^ cells were significantly inhibited in *Brca1* mutant tumors, but monocytes, dendritic cells, and NK cells were dramatically downregulated in *Trp53* mutant tumors (Figure 7A). This suggests that inflammasome inhibition may be applicable in *Brca1* mutant tumor, but not in *TP53* mutant tumor.

**Figure 8:**
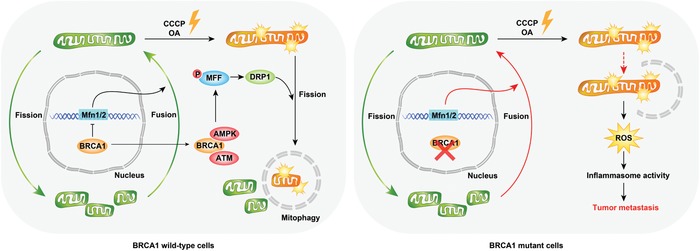
Schematic model for the mechanism by which BRCA1 is essential for mitophagy. BRCA1 is required to maintain mitophagy through involvement in mitochondrial fission and fusion. BRCA1 deficiency then blocks mitophagy, with the accumulation of damaged mitochondria as well as inflammation, involved in tumorigenesis and cancer metastasis.

In summary, we provide the following model for the essential role of BRCA1 in promoting stress‐induced mitophagy to maintain a healthy mitochondrial network at the outer membrane of the mitochondrion (**Figure** **8**). This function occurs through regulating both mitochondrial fusion and fission. We demonstrated that 1) BRCA1 negatively regulates the key components of mitochondrial fusion machinery of MFN1/2 expression under normal conditions; 2) BRCA1 promotes AMPK‐induced DRP1‐MFF activation through mediating mitochondrial translocation of ATM and AMPKα under stress conditions; 3) BRCA1 deficiency impairs stress‐induced mitophagy through blocking ATM‐AMPK‐DRP1‐mediated mitochondrial fission. Excessive mitochondrial fusion leads to elongated mitochondria and prevents the formation of a healthy mitochondrial network in BRCA1 deficient cells, leading to blocked mitophagy; 4) BRCA1 deficiency triggers NLRP3 inflammasome activation and provides a tumor‐associated microenvironment that facilitates tumor proliferation and metastasis. Inhibition of inflammasome activity can serve as a therapeutic approach to fight this deadly disease.

## 4 Experimental Section

##### Animals and Treatments

All animal experiments were approved by the University of Macau Animal Ethics Committee under the protocol (UMAEC‐050‐2015). PDX models of breast cancer were purchased from The Jackson Laboratory (TM00096, *Brca1* WT; TM00089, *Brca1* MT). *Brca1^flox/flox^; MMTV‐Cre* mice were generated in the previous study[qv: 17] and *Trp53^flox/flox^; MMTV‐Cre* mice were generated by crossing *Trp53^flox/flox^* mice[qv: 49] with *MMTV‐Cre* mice. For RNA‐Seq analysis, mammary glands and tumors were collected from female *Brca1^flox/flox^; MMTV‐Cre* mice or *Trp53^flox/flox^; MMTV‐Cre* mice, and mammary glands from female *Brca1^flox/flox^* mice as control group. For tumor recurrence and metastasis experiment, *Brca1^flox/flox^; MMTV‐Cre* mice with mammary tumors (diameter around 1 cm) were i.p. injected with phosphate‐buffered saline (PBS) or NLRP3 inflammasome inhibitor Glibenclamide (GLI, 5 mg kg^−1^) one day before surgery, and mice underwent surgery to remove primary tumors. Then, the mice were administrated with PBS or GLI by continuous injection every three days for five times, and tumor relapse and metastasis were examined.

##### Plasmid

The lentiviral shRNA plasmid pLKO.1 targeting BRCA1 (clone ID TRCN0000039833, TRCN0000039837, and TRCN0000010305), DRP1 (TRCN0000318424), ATM (TRCN0000010299), LKB1 (TRCN0000000408), CaMMKβ (TRCN0000002299), and shRNA control plasmid were purchased from Sigma. MBP‐BRCA1 expressing vector was constructed in previous study.[qv: 50] Mfn1 and Mfn2 luciferase reporters were requested from Dr. Martin Lidell. Six BRCA1 fragments expressing vector were requested from Dr. Rong Li. pCHAC‐mt‐mKeima and pBMN‐mCherry‐Parkin were gifts from Richard Youle (Addgene plasmid # 72342 and # 23956); pRK5‐HA‐Parkin was a gift from Ted Dawson (Addgene plasmid # 17613); pBABEpuro GFP‐LC3 was a gift from Jayanta Debnath (Addgene plasmid # 22405); pLV‐mitoDsRed was a gift from Pantelis Tsoulfas (Addgene plasmid # 44386); LAMP1‐mRFP‐FLAG was a gift from David Sabatini (Addgene plasmid # 34611); pLenti.CMV/TO_PRKAA1 and pLenti.CMV/TO_PRKAA2 were gifts from Reuben Shaw (Addgene plasmid # 74446/74447).

##### Cell Culture and Virus Infection

Hela, 293T, MDA‐MB‐231, MCF7, THP‐1, and MCF 10A cell lines were obtained from the American Type Culture Collection (ATCC). BRCA1 mutant HCC1937 cell line and its derivative reconstituted with full length BRCA1 were gifts from Dr. Junjie Chen.[qv: 51] All cell lines except THP‐1 and MCF 10A cells were grown in dulbecco's modified eagle medium (DMEM) (Thermo Fisher Scientific, 11965118) supplemented with 10% fetal bovine serum (FBS) (Thermo Fisher Scientific, 26140079). THP‐1 cells were cultured with ATCC‐formulated RPMI‐1640 Medium (ATCC, 30‐2001) supplemented with 10% FBS and 0.05 × 10^−3^ m 2‐mercaptoethanol. MCF10A immortalized mammary epithelial cells were cultured with DMEM/F12 (1:1; Thermo Fisher Scientific, 11330057) supplemented with 5% horse serum (Thermo Fisher Scientific, 16050122), 0.5 µg mL^−1^ hydrocortisone (Sigma, H0888), 20 ng mL^−1^ epidermal growth factor (EGF) (Thermo Fisher Scientific, PHG0311L), 10 µg mL^−1^ insulin (Sigma, I1882), and 100 ng mL^−1^ cholera toxin (Sigma, C8052). Primary MEFs were isolated from E13.5–14.5 Flag‐tagged *Brca1*, *Brca1^flox/flox^* or *Brca1^flox/flox^*;*Tam‐Cre* embryos using a standard procedure and maintained in DMEM with 15% FBS. Lentivirus and Retrovirus infection were performed as described.[qv: 52] Adenoviruses expressing Cre recombinase and GFP (Ad‐Cre) or GFP alone (Ad‐GFP) were purchased from Vector Development Laboratory, Baylor College of Medicine, Houston.

##### Transfection and Luciferase Assay

The transfections were carried out with Lipofectamine 3000 (Thermo Fisher Scientific, L3000015) according to the manufacturer's instructions. For luciferase assay, cells were harvested after 24 h post‐transfection and luciferase activity was assayed using a dual luciferase reporter assay system (Promega, Madison, WI). Renilla luciferase activity was used for normalization.

##### Immunofluorescence Staining

The cells were fixed with 4% Paraformaldehyde and then stained with corresponding antibodies using methods described previously.[qv: 52] Deparaffinized sections from primary and recurrent tumors were cooked with Retriever (Electronic Microscopy Science, 62700–10) in Buffer A (citrate buffer, pH 5.0) followed by staining with antibody against F4/80. Images were acquired using Nikon A1R Confocal System or Olympus IX83 Inverted Microscope, and then fluorescent signal intensity, area, and colocalization of signals were analyzed by ImageJ software (National Institutes of Health (NIH), Bethesda). The antibodies used for immunofluorescence staining are listed in Table S2 in the Supporting Information.

##### Mitophagy Treatment and Analysis

Mitophagy treatment and analysis were performed as described previously.[qv: 19] Briefly, mCherry‐Parkin or HA‐Parkin stably expressing Hela cells were established (Hela‐mCherryParkin or Hela‐HA‐Parkin) and infected with lentivirus shCtrl or shBRCA1 for 48 h, and then the cells were treated with 10 × 10^−6^ m carbonyl cyanide *m*‐chlorophenyl hydrazone (CCCP, Sigma‐Aldrich) or 10 × 10^−6^ m oligomycin (Sigma‐Aldrich) combined with 5 × 10^−6^ m antimycin A (Sigma‐Aldrich) for the indicated time points. MEFs were infected with Ad‐Cre or Ad‐GFP for 48 h and then treated with 30 × 10^−6^ m CCCP for the indicated time points. For 24 h treatment, Z‐VAD‐FMK (Sigma‐Aldrich) was supplemented to prevent cells from apoptosis induced by mitochondrial stress. To calculate the percentage of mtDNA stain remaining, the area of total cellular DNA (Area_tDNA_) and nuclear DNA (Area_nDNA_) were determined by anti‐DNA antibody and 4′,6‐diamidino‐2‐phenylindole (DAPI) staining, respectively. mtDNA staining area was calculated by using the following formula: Area_tDNA_‐Area_nDNA_/*n*, in which *n* is cell number in a field, then the mtDNA stain remaining after treatment was normalized by mtDNA staining area without treatment. For mt‐mKeima assay, mt‐mKeima were stably expressed in MEFs by lentivirus transduction, then treated with 4‐HT for 48 h to induce BRCA1 KD. After CCCP treatment, mt‐mKeima were measured on Nikon A1R Confocal System using dual‐excitation measurements at 488 nm (pH 7) and 561 nm (pH 4) lasers with 700/75 nm emission filters. The fluorescence intensity were analyzed using NIS‐Elements software and the ratio of fluorescence intensity between 561 and 488 nm was calculated.

##### Time‐Lapse Microscopy

To visualize mitochondria, Hela‐HA‐Parkin were infected with lentivirus‐MitoDsRed and the fluorescent cells were sorted by flow cytometry (BD FACSAria III). Then, the cells were infected with shCtrl or shBRCA1 lentivirus and seeded in confocal glass‐bottom 6‐well plates (SPL Life Sciences, 30206). Imaging was started immediately after addition of CCCP on Olympus IX83 Inverted Microscope at 37 °C and 5% CO_2_. The images were acquired every 5 or 10 min for 90 min and then were exported as uncompressed AVI sequences at one frame per second.

##### Immunoprecipitation and Western Blot Analysis

For western blot analysis, cells were lysed in radioimmunoprecipitation assay (RIPA) buffer (10 × 10^−3^ m Tris‐HCl pH 8.0, 150 × 10^−3^ m NaCl, 1% Triton X‐100, 1% deoxycholate acid, 0.1% SDS, and protease inhibitor cocktail) after PBS washing. For immunoprecipitation, cells were lysed with lysis buffer (20 × 10^−3^ m Tris‐HCl pH 7.5, 150 × 10^−3^ m NaCl, 10 × 10^−3^ m EDTA, 10 × 10^−3^ m EGTA, 1% NP‐40, and protease inhibitor cocktail) at 24 h post‐transfection, and then the cell lysate was immunoprecipitated by corresponding antibodies or control IgG. After washing, the precipitates were analyzed by Western blot. For λPPase treatment, 1000 units of λPPase (New England Biolabs, P0753), 50 µL 10 × NEBuffer for Protein MetalloPhosphatases, and 1 × 10^−3^ m MnCl_2_ were added into the cell lysate (400 µL) and incubated at 30 °C for 30 min. Western blot was carried out by ChemiDoc MP Imaging System (BIO‐RAD) with correspondent antibodies. The band intensity was quantified using ImageJ, and the numbers under immunoblots represent intensity relative to the first band. Relative quantification was achieved by normalization to the level of β‐actin. Antibodies for immunoprecipitation and western blot are listed in Table S2 in the Supporting Information.

##### Quantitative Real‐Time PCR

Total RNA was isolated with Trizol reagent (Thermo Fisher Scientific) according to the manufacturer's instructions. Reverse transcription was performed using QuantiTect Reverse Transcription Kit (QIAGEN, 205313). Real‐time PCR reactions were performed using FastStart Universal SYBR Green Master (Roche, 4913850001) on QuantStudio 7 Flex Real‐Time PCR System (Thermo Fisher Scientific). Relative quantification was achieved by normalization to the amount of 18S. Primers used for real‐time PCR are listed in Table S3 in the Supporting Information.

##### RNA‐Seq and Data Processing

Total RNA from mammary glands and tumors was processed using RNeasy mini kit (QIAGEN, 74106), and RNA concentration and integrity were measured using the Agilent 2100 Bioanalyzer (Agilent Technologies). cDNA libraries were prepared from RNA starting material (RNA integrity number values > 7.0), using NEBNext Ultra RNA Library Prep Kit for Illumina (New England Biolabs, E7530) according to the manufacturer's instructions, and library quality was checked on Agilent 2100 Bioanalyzer. Total 13 libraries generated from WT MG (two samples), *Brca1* MT MG (two samples), *Brca1* MT tumors (four samples), *Trp53* MT MG (two samples), and *Trp53* MT tumors (three samples) were paired‐end sequenced by HiSeq 2500 (Illumina) in Genomics, Bioinformatics and Single Cell Core, Faculty of Health Science, University of Macau. The quality of the sequencing data was analyzed by using FastQC (version 0.11.5) and raw reads with low quality were removed using Trim Galore (version 0.4.4) prior to analysis of the data. The criteria of removing low quality reads were set as: quality Phred score less than 28 and reads containing adaptor sequences. All the trimmed reads were mapped to reference mouse genome (mm10, GRCm38) by using STAR (version 020201) and Subjunc (version 1.5.3), and the mapped counts were extracted using featureCount from Subread package (version 1.5.3). Subsequently, read count data containing 47594 genes with raw reads were preprocessed by filtering out genes with zero read counts across different samples within cohort. After filtering, 31880 genes remain in per sample. The read count data were normalized to produce transcripts per kilobase million (TPM‐counts per length of transcript (kb) per million reads mapped), which can be used for downstream differential expression analysis. Pathway enrichment analysis was performed using the in‐house R code. All data processing were completed in shell high performance computing (HPC) command line environment. Raw RNA‐Seq data files are available at the NCBI Sequence Read Archive (SRA, PRJNA604672) https://www.ncbi.nlm.nih.gov/bioproject/PRJNA604672.

##### Protein Pull‐Down and Mass Spectrometry Analysis

BRCA1 pull‐down was performed according to the method described previously.[qv: 50,53] Briefly, 293T cells were transfected with MBP‐BRCA1 or control MBP vector. Forty eight hours post‐transfection, these cells were harvested and lysed with lysis buffer containing 0.1% NP‐40, 25 × 10^−3^ m Tris‐HCl (pH 7.5), 150 × 10^−3^ m NaCl, 10% glycerol, 2 × 10^−3^ m MgCl_2_, 0.1 × 10^−3^ m ZnCl_2_, 0.1 × 10^−3^ m EDTA, 1 × 10^−3^ m dithiothreitol (DTT), protease inhibitor cocktail, and phosphatase inhibitor PhoSTOP (Roche). The lysate was sonicated and centrifuged at 14 000 rpm at 4 °C for 10 min. The supernatants were added into Amylose resin (New England Biolabs, E8021S) and rotated for 4 h at 4 °C. After washing four times with lysis buffer, bound proteins were resolved by 4–12% NuPAGE, stained with Coomassie blue, and analyzed via in‐gel digestion followed by liquid chromatography‐mass spectrometry using a linear ion‐trap mass spectrometer (LTQ, ThermoElectron). The LTQ was operated in a data‐dependent mode in which the seven most abundant peptide molecular ions in every mass spectrometry (MS) scan were sequentially selected for fragmentation and acquisition of MS^2^ spectra. Tandem mass spectra were searched against the human protein database using SEQUEST software (ThermoFinnigan). The protein–protein interaction network was built up by STRING (https://string-db.org/)

##### Inflammasome Activity Analysis

THP‐1 cells were differentiated for 3 h with 1 × 10^−6^ m phorbol 12‐myristate 13‐acetate (PMA) (Sigma‐Aldrich), washed, and then primed by lipopolysaccharides (LPS) (200 ng mL^−1^, Sigma‐Aldrich) for 3 h following by CCCP (5 × 10^−6^ m) or Nigericin (10 × 10^−6^ m, Sigma‐Aldrich) treatment for 5 or 1 h, respectively. MCF10A cells were primed by LPS (200 ng mL^−1^) for 3 h following by CCCP or nigericin treatment for 18 h or 5 h, respectively. After treatment, cell lysis and cell culture supernatants were collected for analyzing inflammasome activity. Cell culture supernatants were assayed for IL‐1β with ELISA Ready‐SET‐Go! Kit (Thermo Fisher Scientific, 88‐7261‐86) according to the manufacturer's instructions. For measurement of extracellular cleaved IL‐1β and caspase‐1 by Western Blot, cell culture supernatants were precipitated by the addition of an equal volume of methanol and 0.25 volumes of chloroform as described previously.[qv: 54] The supernatant/methanol/chloroform mixtures were vortexed and then centrifuged for 10 min at 20 000 × *g*. The upper phase was discarded and 500 µL methanol was added to the interphase. This mixture was centrifuged for 10 min at 20 000 × *g* and the protein pellet was dried at 55 °C.

##### Reactive Oxygen Species and Mitochondrial Membrane Potential Analysis by Fluorescence‐activated Cell Sorting (FACS)

For ROS measurement, intracellular H_2_O_2_ was measured by H_2_DCFDA (10 × 10^−6^ m, Thermo Fisher Scientific) and mitochondrial ROS was determined by MitoSOX (5 × 10^−6^ m, Thermo Fisher Scientific). In brief, cells were performed with CCCP (10 × 10^−6^ m) for 18 h and then incubated with H_2_DCFDA or MitoSOX for 30 min according to manufacturer's instructions, then cells were collected and analyzed by flow cytometry. For mitochondrial membrane potential analysis, the cells were stained with both Mitotracker Green (200 × 10^−9^ m, Thermo Fisher Scientific) and tetramethylrhodamine ethyl ester perchlorate (TMRE) (100 × 10^−9^ m, Thermo Fisher Scientific) after CCCP treatment for 18 h, then mitochondrial membrane potential per unit of mitochondrial mass on a single cell was assessed by flow cytometry.

##### Mitochondrial Isolation

MCF7 or MEF cells were treated with CCCP (10 or 30 × 10^−6^ m, respectively) for the indicated time points. Mitochondrial isolation were performed by Mitochondria Isolation Kit according to the manufacturer's instructions (Abcam, ab110171). For MCF7 cells, isolated mitochondria were treated by proteinase K (0.8 mg mL^−1^) alone or proteinase K combined with digitonin (0.2 mg mL^−1^) and 1% SDS, then harvested for immunoblot.

##### Tumor Digestion and Flow Cytometry

Tumors were isolated, finely minced, and digested with Digestion I at 37 °C for 4 h. After cells were spun down, they were treated with Digestion II for 5 min. Digested tumor cells were washed with Hanks solution and lysed with red blood cell (RBC) Lysis Solution. Cells were resuspended in Flow Cytometry Staining Buffer (ThermoFisher Scientific) and incubated with antibodies (Table S2, Supporting Information) for 1 h on ice. Then, the cells were washed and resuspended by Flow Cytometry Staining Buffer, and analyzed by flow cytometry (BD FACSAria III). Signal threshold definition was defined using all‐stained, unstained and isotype controls. Gating strategy is shown in Figure S9B in the Supporting Information. Digestion I: DMEM/F12 supplemented with 5% FBS, 500 ng mL^−1^ hydrocortisone, 10 ng mL^−1^ EGF, 20 ng mL^−1^ cholera toxin, 300 U mL^−1^ collagenase III, 100 U mL^−1^ hyaluronidase; Digestion II: 5 mg mL^−1^ dispase II, 0.1 mg mL^−1^ deoxyribonuclease.

##### Clinical Data from The METABRIC

METABRIC breast cancer normalized transcriptome data and mutation file with clinical information were downloaded from the cBioPortal (https://www.cbioportal.org).[qv: 37,55] Thirty‐six *BRCA1* mutant cancers with single‐nucleotide variant or indel mutation and 34 *TP53* mutant cancers with R175 mutation were found (Table S4, Supporting Information). Thus, a total of 24368 genes were used for GSEA and macrophage polarization analysis using *TP53* mutant group as control.

##### Gene Set Enrichment Analysis (GSEA) and Macrophage Polarization Analysis

GSEA was performed using the R package clusterProfiler.[qv: 56] Briefly, the enrichment score (ES) was calculated as the maximum deviation from zero of the weighted fraction of genes present minus the fraction not present up to a given index in a gene expression matrix ordered by phenotype correlation. The statistical significance (nominal *P* value) of the ES of a gene set was estimated using an empirical gene‐based permutation test. The normalized enrichment score was calculated by creating 1000 permutations of the ES and scaling the observed ES by the mean score of the permutations. GSEA was applied to selected gene sets to test their enrichment in given datasets including mitochondrion organization (GO:0007005), inflammatory response (GO:0006954), and activated CD8 T cell.[qv: 57] To analyze macrophage polarization, macrophage signature genes were obtained including 56/37 (mouse/human) M1 marker genes and 33/29 (mouse/human) M2 marker genes (Table S5, Supporting Information) according to previous study.[qv: 58] Macrophage polarization analysis was performed as described previously.[qv: 59] The expression of the marker genes were first normalized across patients, and a Student's *t*‐test score comparing the expression of macrophage M2 marker genes to macrophage M1 marker genes was considered as M2‐M1 score. The score greater than zero reflects samples with M2 macrophage polarization feature, while the score less than zero reflects samples with M1 macrophage feature.

##### Statistical Analysis

All values are expressed as the mean ± standard error of the mean (SEM) of individual samples. Samples were analyzed by using unpaired two‐tailed *t*‐test or one/two‐way analysis of variance (ANOVA) test. Values of *p* < 0.05 were considered as statistically significant. Correlation was studied by Pearson's correlation test as indicated. Statistical analysis was performed using GraphPad Prism 7.

## Conflict of Interest

The authors declare no conflict of interest.

## Author Contributions

Q.C. and C.‐X.D. conceived and designed the study and wrote the paper. Q.C., H.L., J.B., W.H., L.L., C.P., T.M., and J.X. acquired the data. Q.C., H.L., J.B., W.H., H.W., L.L., C.P., and T.M. developed the methodologies. Q.C., H.L., J.B., W.H., H.W., and L.L. analyzed and interpreted the data. H.L., K.M., and X.X. provided mice.

## Supporting information

Supporting Information

Supplemental Movie 1

Supplemental Movie 2

Supplemental Table 1

Supplemental Table 2

Supplemental Table 3

Supplemental Table 4

Supplemental Table 5

## References

[1] R. L. Siegel , K. D. Miller , A. Jemal , Ca‐Cancer J. Clin. 2019, 69, 7. 30620402 10.3322/caac.21551

[2] W. D. Foulkes , I. E. Smith , J. S. Reis‐Filho , N. Engl. J. Med. 2010, 363, 1938. 21067385 10.1056/NEJMra1001389

[3] G. Bianchini , J. M. Balko , I. A. Mayer , M. E. Sanders , L. Gianni , Nat. Rev. Clin. Oncol. 2016, 13, 674. 27184417 10.1038/nrclinonc.2016.66 PMC5461122

[4] S. Bayraktar , A. M. Gutierrez‐Barrera , D. Liu , T. Tasbas , U. Akar , J. K. Litton , E. Lin , C. T. Albarracin , F. Meric‐Bernstam , A. M. Gonzalez‐Angulo , G. N. Hortobagyi , B. K. Arun , Breast Cancer Res. Treat. 2011, 130, 145. 21830012 10.1007/s10549-011-1711-z PMC4334122

[5] C. X. Deng , Nucleic Acids Res. 2006, 34, 1416. 16522651 10.1093/nar/gkl010 PMC1390683

[6] J. Dine , C. X. Deng , Cancer Metastasis Rev. 2013, 32, 25. 23093327 10.1007/s10555-012-9403-7

[7] J. A. Rodriguez , B. R. Henderson , J. Biol. Chem. 2000, 275, 38589. 10991937 10.1074/jbc.M003851200

[8] J. Jiang , E. S. Yang , G. Jiang , S. Nowsheen , H. Wang , T. Wang , Y. Wang , D. Billheimer , A. B. Chakravarthy , M. Brown , B. Haffty , F. Xia , Cancer Res. 2011, 71, 5546. 21742769 10.1158/0008-5472.CAN-10-3423 PMC12912278

[9] S. Pickles , P. Vigie , R. J. Youle , Curr. Biol. 2018, 28, R170. 29462587 10.1016/j.cub.2018.01.004 PMC7255410

[10] S. L. Archer , N. Engl. J. Med. 2013, 369, 2236. 24304053 10.1056/NEJMra1215233

[11] J. W. Harper , A. Ordureau , J. M. Heo , Nat. Rev. Mol. Cell Biol. 2018, 19, 93. 29358684 10.1038/nrm.2017.129

[12] R. Zhou , A. S. Yazdi , P. Menu , J. Tschopp , Nature 2011, 469, 221. 21124315 10.1038/nature09663

[13] S. Vyas , E. Zaganjor , M. C. Haigis , Cell 2016, 166, 555. 27471965 10.1016/j.cell.2016.07.002 PMC5036969

[14] K. K. Singh , P. C. Shukla , B. Yanagawa , A. Quan , F. Lovren , Y. Pan , C. S. Wagg , H. Teoh , G. D. Lopaschuk , S. Verma , J. Thorac. Cardiovasc. Surg. 2013, 146, 702. 23317938 10.1016/j.jtcvs.2012.12.046

[15] K. C. Jackson , E. K. Gidlund , J. Norrbom , A. P. Valencia , D. M. Thomson , R. A. Schuh , P. D. Neufer , E. E. Spangenburg , J. Lipid Res. 2014, 55, 668. 24565757 10.1194/jlr.M043851 PMC3966701

[16] K. C. Jackson , M. D. Tarpey , A. P. Valencia , M. R. Inigo , S. J. Pratt , D. J. Patteson , J. M. McClung , R. M. Lovering , D. M. Thomson , E. E. Spangenburg , FASEB J. 2018, 32, 3070. 29401626 10.1096/fj.201700464R PMC5956240

[17] X. Xu , K. U. Wagner , D. Larson , Z. Weaver , C. Li , T. Ried , L. Hennighausen , A. Wynshaw‐Boris , C. X. Deng , Nat. Genet. 1999, 22, 37. 10319859 10.1038/8743

[18] H. Katayama , T. Kogure , N. Mizushima , T. Yoshimori , A. Miyawaki , Chem. Biol. 2011, 18, 1042. 21867919 10.1016/j.chembiol.2011.05.013

[19] M. Lazarou , D. A. Sliter , L. A. Kane , S. A. Sarraf , C. Wang , J. L. Burman , D. P. Sideris , A. I. Fogel , R. J. Youle , Nature 2015, 524, 309. 26266977 10.1038/nature14893 PMC5018156

[20] S. Geisler , K. M. Holmstrom , D. Skujat , F. C. Fiesel , O. C. Rothfuss , P. J. Kahle , W. Springer , Nat. Cell Biol. 2010, 12, 119. 20098416 10.1038/ncb2012

[21] M. E. Gegg , J. M. Cooper , K. Y. Chau , M. Rojo , A. H. Schapira , J. W. Taanman , Hum. Mol. Genet. 2010, 19, 4861. 20871098 10.1093/hmg/ddq419 PMC3583518

[22] A. M. van der Bliek , Q. Shen , S. Kawajiri , Cold Spring Harbor Perspect. Biol. 2013, 5, a011072. 10.1101/cshperspect.a011072 PMC3660830 23732471

[23] J. Y. Peng , C. C. Lin , Y. J. Chen , L. S. Kao , Y. C. Liu , C. C. Chou , Y. H. Huang , F. R. Chang , Y. C. Wu , Y. S. Tsai , C. N. Hsu , PLoS Comput. Biol. 2011, 7, e1002212. 21998575 10.1371/journal.pcbi.1002212 PMC3188504

[24] G. Twig , A. Elorza , A. J. Molina , H. Mohamed , J. D. Wikstrom , G. Walzer , L. Stiles , S. E. Haigh , S. Katz , G. Las , J. Alroy , M. Wu , B. F. Py , J. Yuan , J. T. Deeney , B. E. Corkey , O. S. Shirihai , EMBO J. 2008, 27, 433. 18200046 10.1038/sj.emboj.7601963 PMC2234339

[25] M. M. Mihaylova , R. J. Shaw , Nat. Cell Biol. 2011, 13, 1016. 21892142 10.1038/ncb2329 PMC3249400

[26] S. L. Clark , A. M. Rodriguez , R. R. Snyder , G. D. Hankins , D. Boehning , Comput. Struct. Biotechnol. J. 2012, 1, e201204005. 22737296 10.5936/csbj.201204005 PMC3380633

[27] D. P. Silver , D. M. Livingston , Cancer Discovery 2012, 2, 679. 22843421 10.1158/2159-8290.CD-12-0221 PMC3437262

[28] E. Q. Toyama , S. Herzig , J. Courchet , T. L. Lewis Jr. , O. C. Loson , K. Hellberg , N. P. Young , H. Chen , F. Polleux , D. C. Chan , R. J. Shaw , Science 2016, 351, 275. 26816379 10.1126/science.aab4138 PMC4852862

[29] A. Suzuki , G. Kusakai , A. Kishimoto , Y. Shimojo , T. Ogura , M. F. Lavin , H. Esumi , Biochem. Biophys. Res. Commun. 2004, 324, 986. 15485651 10.1016/j.bbrc.2004.09.145

[30] N. Foray , D. Marot , A. Gabriel , V. Randrianarison , A. M. Carr , M. Perricaudet , A. Ashworth , P. Jeggo , EMBO J. 2003, 22, 2860. 12773400 10.1093/emboj/cdg274 PMC156770

[31] S. K. Bunt , P. Sinha , V. K. Clements , J. Leips , S. Ostrand‐Rosenberg , J. Immunol. 2006, 176, 284. 16365420 10.4049/jimmunol.176.1.284

[32] B. Guo , S. Fu , J. Zhang , B. Liu , Z. Li , Sci. Rep. 2016, 6, 36107. 27786298 10.1038/srep36107 PMC5082376

[33] S. G. Brodie , X. Xu , W. Qiao , W. M. Li , L. Cao , C. X. Deng , Oncogene 2001, 20, 7514. 11709723 10.1038/sj.onc.1204929

[34] X. Xu , W. Qiao , S. P. Linke , L. Cao , W. M. Li , P. A. Furth , C. C. Harris , C. X. Deng , Nat. Genet. 2001, 28, 266. 11431698 10.1038/90108

[35] D. Walerych , M. Napoli , L. Collavin , G. Del Sal , Carcinogenesis 2012, 33, 2007. 22822097 10.1093/carcin/bgs232 PMC3483014

[36] A. Mantovani , S. Sozzani , M. Locati , P. Allavena , A. Sica , Trends Immunol. 2002, 23, 549. 12401408 10.1016/s1471-4906(02)02302-5

[37] C. Curtis , S. P. Shah , S. F. Chin , G. Turashvili , O. M. Rueda , M. J. Dunning , D. Speed , A. G. Lynch , S. Samarajiwa , Y. Yuan , S. Graf , G. Ha , G. Haffari , A. Bashashati , R. Russell , S. McKinney , M. Group , A. Langerod , A. Green , E. Provenzano , G. Wishart , S. Pinder , P. Watson , F. Markowetz , L. Murphy , I. Ellis , A. Purushotham , A. L. Borresen‐Dale , J. D. Brenton , S. Tavare , C. Caldas , S. Aparicio , Nature 2012, 486, 346. 22522925 10.1038/nature10983 PMC3440846

[38] P. A. Muller , K. H. Vousden , Cancer Cell 2014, 25, 304. 24651012 10.1016/j.ccr.2014.01.021 PMC3970583

[39] R. Sumpter Jr. , S. Sirasanagandla , A. F. Fernandez , Y. Wei , X. Dong , L. Franco , Z. Zou , C. Marchal , M. Y. Lee , D. W. Clapp , H. Hanenberg , B. Levine , Cell 2016, 165, 867. 27133164 10.1016/j.cell.2016.04.006 PMC4881391

[40] M. D. Tarpey , A. P. Valencia , K. C. Jackson , A. J. Amorese , N. P. Balestrieri , R. H. Renegar , S. J. P. Pratt , T. E. Ryan , J. M. McClung , R. M. Lovering , E. E. Spangenburg , J. Physiol. 2019, 597, 869. 30556208 10.1113/JP276863 PMC6355718

[41] C. Magnard , R. Bachelier , A. Vincent , M. Jaquinod , S. Kieffer , G. M. Lenoir , N. D. Venezia , Oncogene 2002, 21, 6729. 12360400 10.1038/sj.onc.1205915

[42] K. Moreau , E. Dizin , H. Ray , C. Luquain , E. Lefai , F. Foufelle , M. Billaud , G. M. Lenoir , N. D. Venezia , J. Biol. Chem. 2006, 281, 3172. 16326698 10.1074/jbc.M504652200

[43] E. D. Coene , M. S. Hollinshead , A. A. Waeytens , V. R. Schelfhout , W. P. Eechaute , M. K. Shaw , P. M. Van Oostveldt , D. J. Vaux , Mol. Biol. Cell 2005, 16, 997. 15591126 10.1091/mbc.E04-10-0895 PMC545929

[44] A. W. Maniccia , C. Lewis , N. Begum , J. Xu , J. Cui , G. Chipitsyna , K. Aysola , V. Reddy , G. Bhat , Y. Fujimura , B. Henderson , E. S. Reddy , V. N. Rao , J. Cell. Physiol. 2009, 219, 634. 19170108 10.1002/jcp.21708 PMC3693557

[45] V. Tembe , B. R. Henderson , J. Biol. Chem. 2007, 282, 20513. 17510055 10.1074/jbc.M702627200

[46] V. Tembe , E. Martino‐Echarri , K. A. Marzec , M. T. Mok , K. M. Brodie , K. Mills , Y. Lei , A. DeFazio , H. Rizos , E. Kettle , R. Boadle , B. R. Henderson , Cell. Signalling 2015, 27, 1763. 26022179 10.1016/j.cellsig.2015.05.011

[47] J. A. Krall , F. Reinhardt , O. A. Mercury , D. R. Pattabiraman , M. W. Brooks , M. Dougan , A. W. Lambert , B. Bierie , H. L. Ploegh , S. K. Dougan , R. A. Weinberg , Sci. Transl. Med. 2018, 10, eaan3464. 29643230 10.1126/scitranslmed.aan3464 PMC6364295

[48] M. Retsky , R. Demicheli , W. J. Hrushesky , P. Forget , M. De Kock , I. Gukas , R. A. Rogers , M. Baum , V. Sukhatme , J. S. Vaidya , Curr. Med. Chem. 2013, 20, 4163. 23992307 10.2174/09298673113209990250 PMC3831877

[49] S. C. Lin , K. F. Lee , A. Y. Nikitin , S. G. Hilsenbeck , R. D. Cardiff , A. Li , K. W. Kang , S. A. Frank , W. H. Lee , E. Y. Lee , Cancer Res. 2004, 64, 3525. 15150107 10.1158/0008-5472.CAN-03-3524

[50] T. Masuda , X. Xu , E. K. Dimitriadis , T. Lahusen , C. X. Deng , Int. J. Biol. Sci. 2016, 12, 133. 26884712 10.7150/ijbs.14242 PMC4737671

[51] R. Scully , S. Ganesan , K. Vlasakova , J. Chen , M. Socolovsky , D. M. Livingston , Mol. Cell 1999, 4, 1093. 10635334 10.1016/s1097-2765(00)80238-5

[52] Q. Chen , W. Hao , C. Xiao , R. Wang , X. Xu , H. Lu , W. Chen , C. X. Deng , Cell Rep. 2017, 18, 3155. 28355567 10.1016/j.celrep.2017.03.006 PMC9396928

[53] R. H. Wang , X. Xu , H. S. Kim , Z. Xiao , C. X. Deng , Int. J. Biol. Sci. 2013, 9, 934. 24163589 10.7150/ijbs.7529 PMC3807017

[54] T. Fernandes‐Alnemri , J. W. Yu , P. Datta , J. Wu , E. S. Alnemri , Nature 2009, 458, 509. 19158676 10.1038/nature07710 PMC2862225

[55] J. Gao , B. A. Aksoy , U. Dogrusoz , G. Dresdner , B. Gross , S. O. Sumer , Y. Sun , A. Jacobsen , R. Sinha , E. Larsson , E. Cerami , C. Sander , N. Schultz , Sci. Signaling 2013, 6, pl1. 10.1126/scisignal.2004088 PMC4160307 23550210

[56] G. Yu , L. G. Wang , Y. Han , Q. Y. He , OMICS: J. Integr. Biol. 2012, 16, 284. 10.1089/omi.2011.0118 PMC3339379 22455463

[57] P. Charoentong , F. Finotello , M. Angelova , C. Mayer , M. Efremova , D. Rieder , H. Hackl , Z. Trajanoski , Cell Rep. 2017, 18, 248. 28052254 10.1016/j.celrep.2016.12.019

[58] K. A. Jablonski , S. A. Amici , L. M. Webb , D. Ruiz‐Rosado Jde , P. G. Popovich , S. Partida‐Sanchez , M. Guerau‐de‐Arellano , PLoS One 2015, 10, e0145342. 26699615 10.1371/journal.pone.0145342 PMC4689374

[59] The Cancer Genome Atlas Research Network , Nature 2011, 474, 609.21720365

