# Supplementary material for: BRCA1 Deficiency Impairs Mitophagy and Promotes Inflammasome Activation and Mammary Tumor Metastasis
Source: Adv Sci (Weinh). 2020 Feb 14;7(6):1903616. doi: 10.1002/advs.201903616 (PMC7080549; doi:10.1002/advs.201903616)
Supplement: Supplementary file 1 — Supporting Information [file ADVS-7-1903616-s001.pdf]

Copyright WILEY-VCH Verlag GmbH & Co. KGaA, 69469 Weinheim, Germany, 2020.

ADVANCED SCIENCE Open Access

Supporting Information

for *Adv. Sci.,* DOI: 10.1002/advs.201903616

BRCA1 Deficiency Impairs Mitophagy and Promotes Inflammasome Activation and Mammary Tumor Metastasis

Qiang Chen, Josh Haipeng Lei, Jiaolin Bao, Haitao Wang, Wenhui Hao, Licen Li, Cheng Peng, Takaaki Masuda, Kai Miao, Jun Xu, Xiaoling Xu, and Chu-Xia Deng**

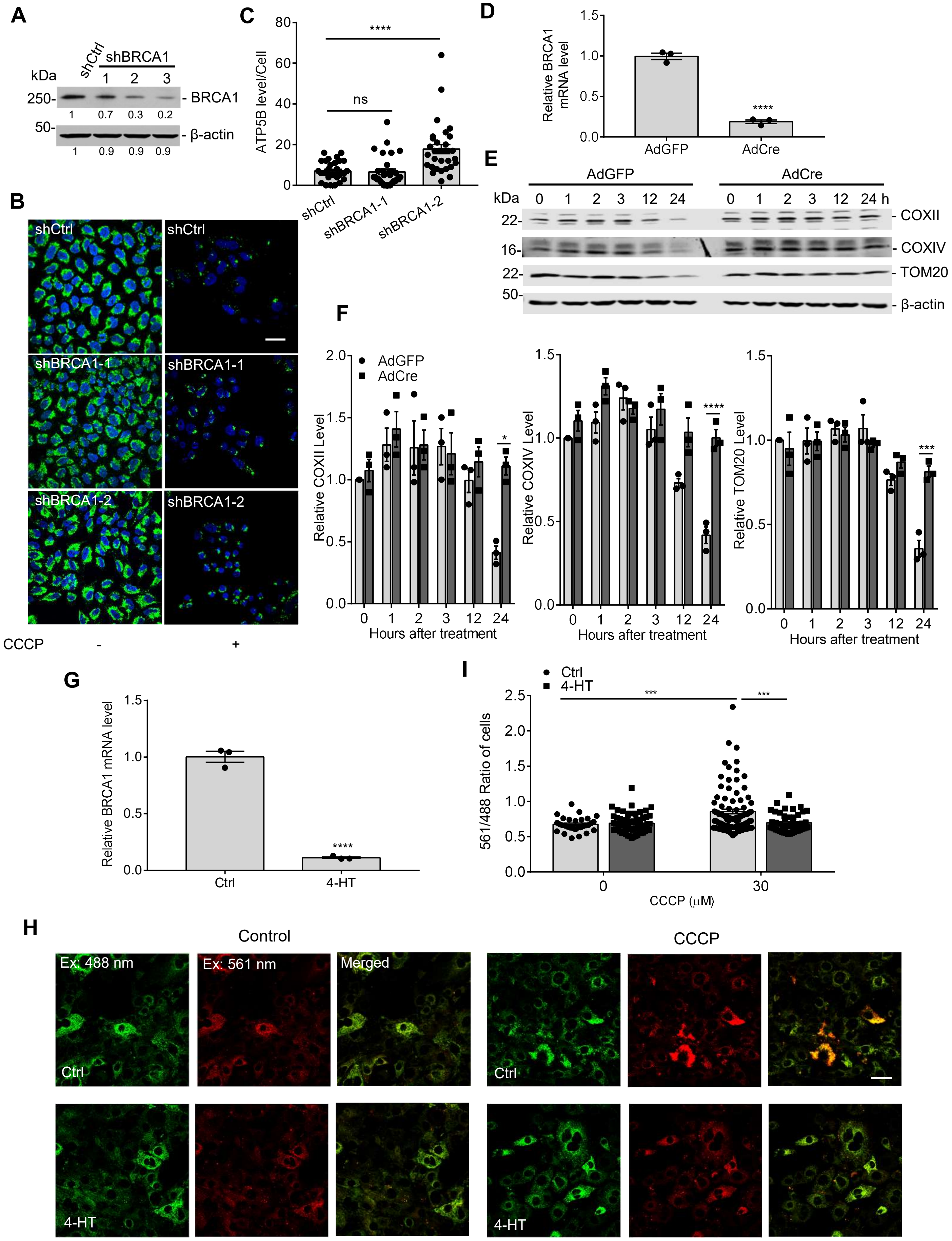
A
kDa
shCtrl
shBRCA1
1 2 3
250-
- BRCA1
1 0.7 0.3 0.2
50-
- β-actin
1 0.9 0.9 0.9
B
shCtrl
shBRCA1-1
shBRCA1-2
CCCP - +
C
ATP5B level/Cell
****
ns
shCtrl
shBRCA1-1
shBRCA1-2
D
Relative BRCA1 mRNA level
****
AdGFP
AdCre
E
AdGFP
AdCre
kDa 0 1 2 3 12 24 0 1 2 3 12 24 h
22-
- COXII
16-
- COXIV
22-
- TOM20
50-
- β-actin
F
AdGFP
AdCre
Relative COXII Level
Relative COXIV Level
Relative TOM20 Level
Hours after treatment
G
Relative BRCA1 mRNA level
****
Ctrl
4-HT
I
Ctrl
4-HT
***
561/488 Ratio of cells
CCCP (μM)
H
Control
CCCP
Ex: 488 nm
Ex: 561 nm
Merged
Ctrl
4-HT

Figure S1. Loss of BRCA1 impairs stress-induced mitophagy. A) Immunoblot analysis of BRCA1 in Hela-mCherryParkin with control shRNA or different BRCA1 shRNA. **B)** Analysis of mitophagy activities in shCtrl and shBRCA1 Hela-mCherryParkin under CCCP treatment by clearance of ATP5B. Scale bar, 20 µm. **C)** Quantification for ATP5B levels after CCCP treatment (more than 30 cells were counted per group). **D)** The mRNA levels of BRCA1 in *Brca1$^{flox/flox}$* MEFs infected with AdGFP or AdCre. **E)** Immunoblot analysis of COXII, COXIV and TOM20 in *Brca1$^{flox/flox}$* MEFs infected with AdGFP or AdCre treated with CCCP. **F)** Quantification of COXII, COXIV and TOM20 levels in (D), which normalized by β-actin level (n = 3 per group). **G)** The mRNA levels of BRCA1 in *Brca1$^{flox/flox}$;Tam-Cre* MEFs with or without 4-HT treatment. **H, I)** Mitophagy activity indicated by mt-mKeima in *Brca1$^{flox/flox}$;Tam-Cre* MEFs. (H) Dual-excitation imaging (488/561 nm) of mt-mKeima. Scale bar, 50 µm. (I) Quantification of fluorescence intensity from (H) (more than 50 cells were counted per group). A high ratio (561/488) of puncta indicates a low pH area. The cells were treated with or not CCCP (30 µM). Data represent the mean ± SEM and are representative of three independent experiments. Significant differences were determined by one-way (C) or two-way (F, I) ANOVA with Tukey multiple comparison testing or unpaired two-tailed *t* test (D and G). *$p < 0.05$, ***$p < 0.001$, ****$p < 0.0001$.

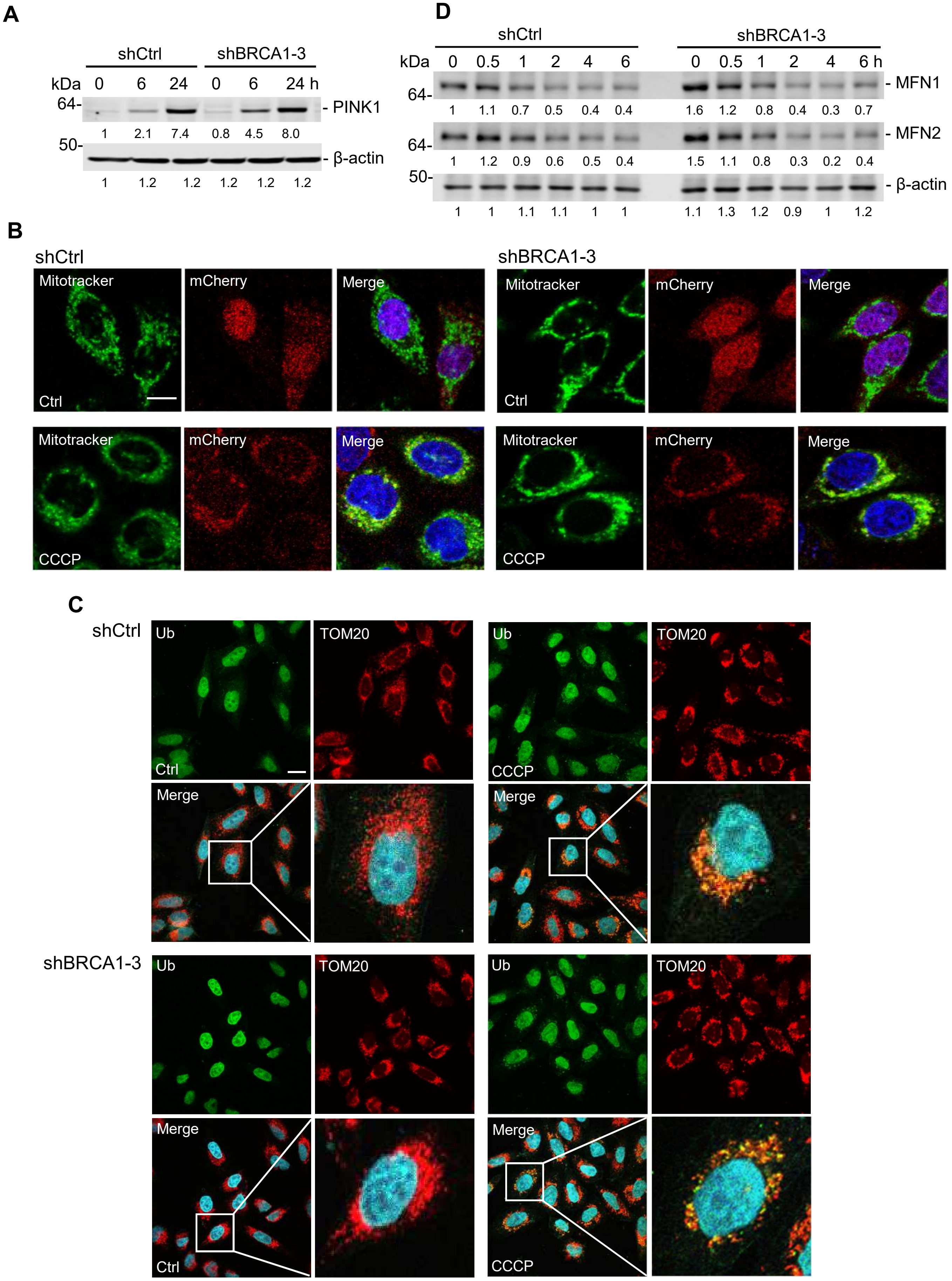

A
shCtrl
shBRCA1-3
kDa 0 6 24 0 6 24 h
64-
- PINK1
1 2.1 7.4 0.8 4.5 8.0
50-
- β-actin
1 1.2 1.2 1.2 1.2 1.2
D
shCtrl
shBRCA1-3
kDa 0 0.5 1 2 4 6 0 0.5 1 2 4 6 h
64-
- MFN1
1 1.1 0.7 0.5 0.4 0.4 1.6 1.2 0.8 0.4 0.3 0.7
64-
- MFN2
1 1.2 0.9 0.6 0.5 0.4 1.5 1.1 0.8 0.3 0.2 0.4
50-
- β-actin
1 1 1.1 1.1 1 1 1.1 1.3 1.2 0.9 1 1.2
B
shCtrl
shBRCA1-3
Mitotracker
mCherry
Merge
Ctrl
CCCP
C
shCtrl
shBRCA1-3
Ub
TOM20
Merge
Ctrl
CCCP

Figure S2. BRCA1 deficiency has no effect on CCCP induced PINK1/Parkin pathway activation. A) Immunoblot analysis of PINK1 in shCtrl and shBRCA1 Hela cells after CCCP (10 μM) treatment for indicated time points. **B)** shCtrl and shBRCA1 Hela-mCherryParkin were treated by CCCP for 1 h, and then stained by MitoTracker Green for labeling mitochondria. Hochest33258, DNA-binding dye. Scale bar, 10 μm. **C)** The ubiquitin level on mitochondria in shCtrl and shBRCA1 Hela-HA-Parkin with or without CCCP (10 μM) treatment for 3 h, as measured by immunostaining for ubiquitin (Ub). Mitochondria were labeled by TOM20 immunostaining. Scale bar, 10 μm. **D)** Immunoblot analysis of MFN1 and MFN2 levels in Hela-HA-Parkin shCtrl and shBRCA1 cells under CCCP treatment for indicated time points. Data are representative of at least two independent experiments.

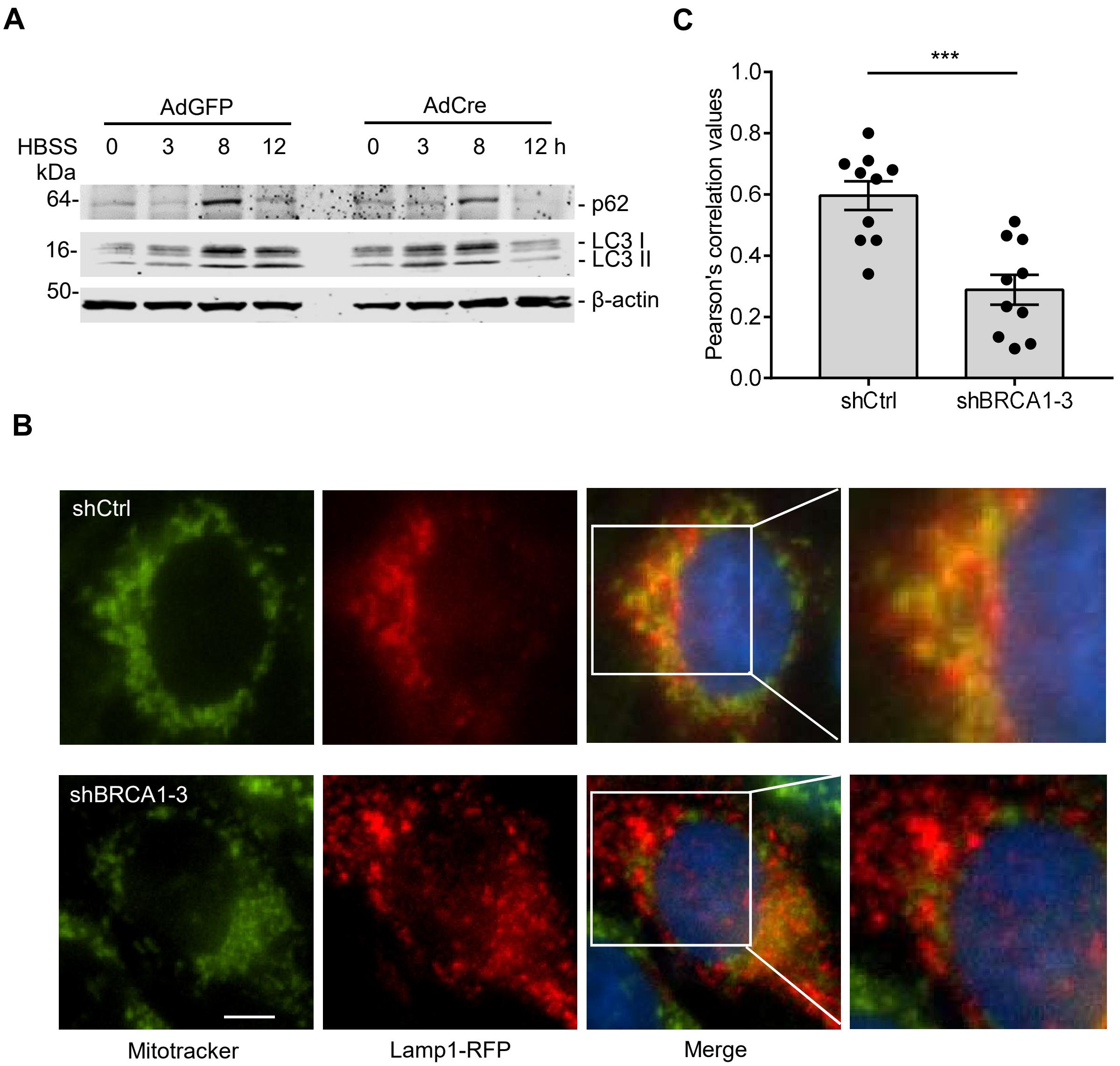

Figure S3. BRCA1 is required for mitophagy, but not for general autophagy. A) Immunoblot analysis of p62 and LC3 in *Brca1*$^{flox/flox}$ MEFs infected with AdGFP or AdCre under starvation (HBSS treatment) for indicated times. **B)** The co-localization of lysosomes with mitochondria in Hela-HA-Parkin shCtrl and shBRCA1 cells under CCCP (10 μM) treatment for 6 h. Lysosomes and mitochondira were marked with LAMP1-RFP and MitoTracker Green, respectively. Scale bar, 5 μm. **C)** Pearson's coefficient is shown as the quantification of lysosomes co-localized with mitochondria per cell in (B) (10 fields were counted per group). Data represent the mean ± SEM and are representative of at least two independent experiments. Significant differences were determined by unpaired two-tailed *t* test (C) ****p* < 0.001.

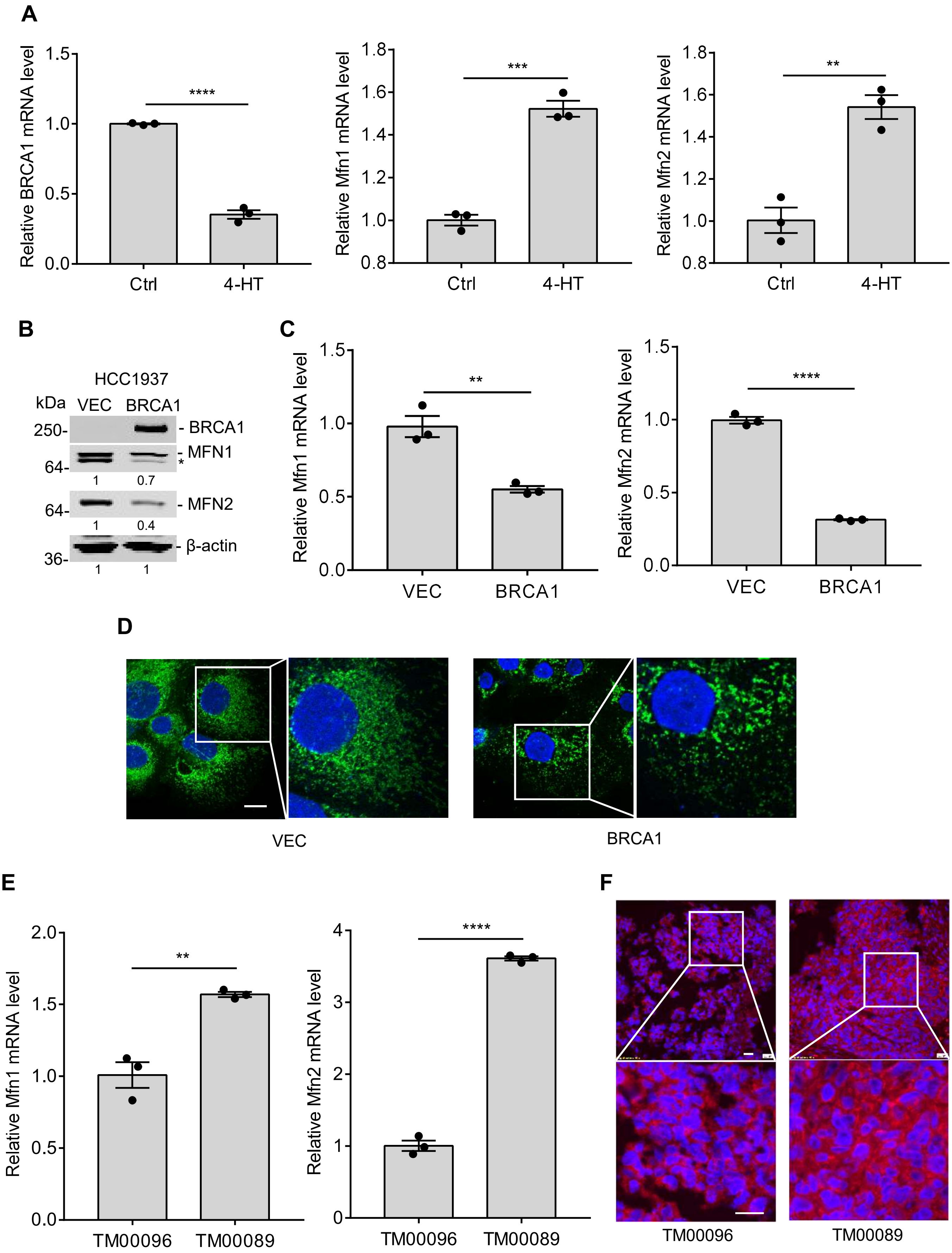
A
Relative BRCA1 mRNA level
1.5
1.0
0.5
0.0
****
Ctrl
4-HT
Relative Mfn1 mRNA level
1.8
1.6
1.4
1.2
1.0
0.8
***
Ctrl
4-HT
Relative Mfn2 mRNA level
1.8
1.6
1.4
1.2
1.0
0.8
**
Ctrl
4-HT
B
HCC1937
kDa
VEC
BRCA1
250-
- BRCA1
- MFN1
*
64-
1
0.7
- MFN2
64-
1
0.4
- β-actin
36-
1
1
C
Relative Mfn1 mRNA level
1.5
1.0
0.5
0.0
**
VEC
BRCA1
Relative Mfn2 mRNA level
1.5
1.0
0.5
0.0
****
VEC
BRCA1
D
VEC
BRCA1
E
Relative Mfn1 mRNA level
2.0
1.5
1.0
0.5
0.0
**
TM00096
TM00089
Relative Mfn2 mRNA level
4
3
2
1
0
****
TM00096
TM00089
F
TM00096
TM00089

Figure S4. BRCA1 regulates MFN1/2 mediated-mitochondrial fusion. A) The mRNA levels of BRCA1, Mfn1 and Mfn2 in *Brca1*$^{flox/flox}$; *Tam-Cre* MEFs with or without 4-HT (5 µM) treatment, as measured by real-time PCR (n = 3 per group). **B)** Immunoblot analysis of BRCA1, MFN1 and MFN2 in HCC1937 cells stably transfected with empty vector (VEC) or BRCA1 expressing vector (BRCA1). Asterisk (*) indicates MFN2 band. **C)** The mRNA levels of Mfn1 and Mfn2 in VEC and BRCA1 HCC1937 cells measured by real-time PCR (n = 3 per group). **D)** Mitochondrial morphology of VEC and BRCA1 HCC1937 cells stained by MitoTracker Green. Scale bar, 10 µm. **E)** The mRNA levels of Mfn1 and Mfn2 in breast cancer PDX models (TM00096, *BRCA1* WT; TM00089, *BRCA1* MT) cells measured by real-time PCR (n = 3 per group). **F)** Representative images of immunostained MFN1 in breast cancer PDX models. Scale bar, 20 µm. Data represent the mean ± SEM and are representative of at least two independent experiments. Significant differences were determined by unpaired two-tailed *t* test. $^{**}p < 0.01$, $^{***}p < 0.001$, $^{****}p < 0.0001$.

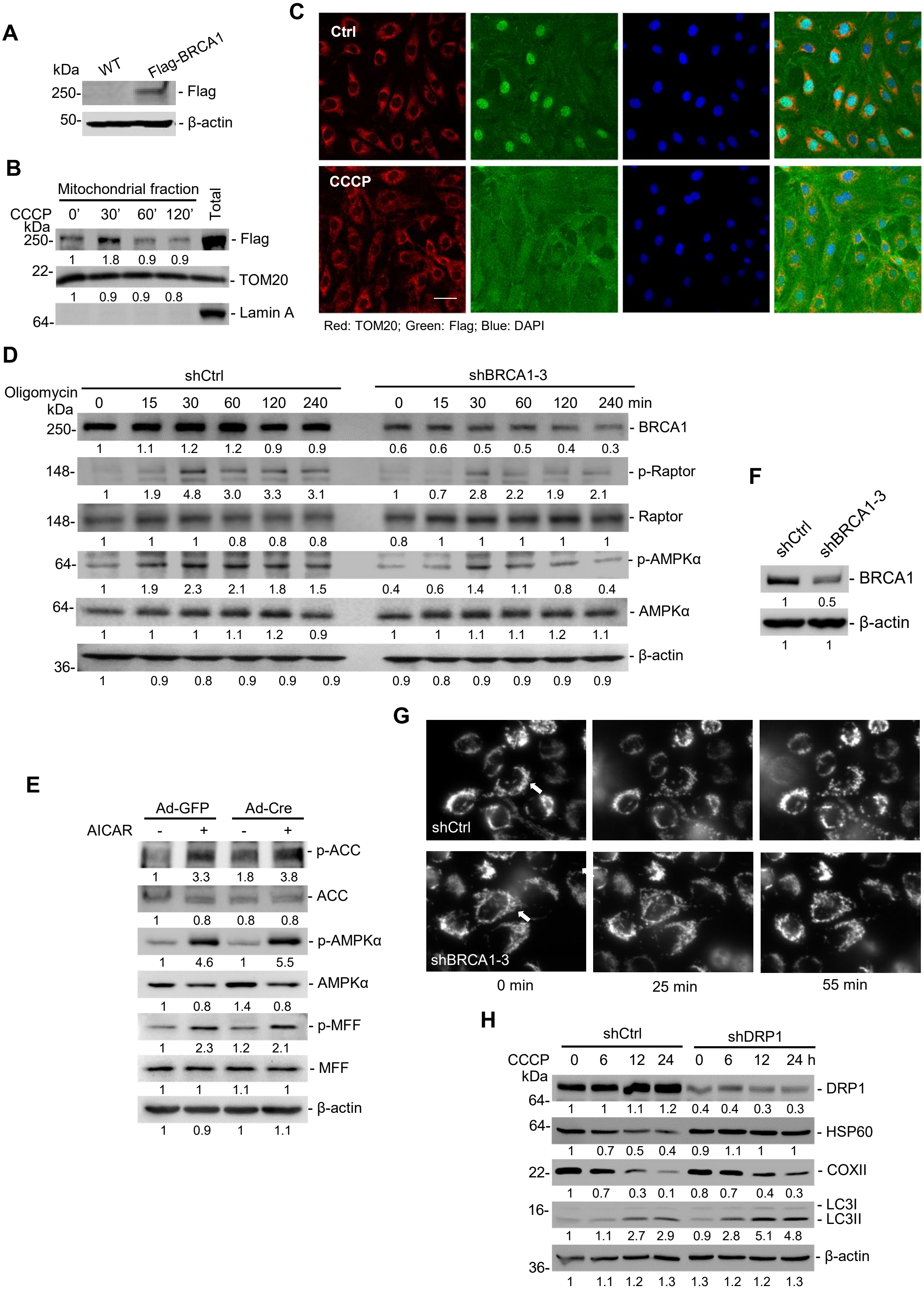
A
kDa
WT
Flag-BRCA1
250-
- Flag
50-
- β-actin
B
Mitochondrial fraction
Total
CCCP 0' 30' 60' 120'
kDa
250-
- Flag
1 1.8 0.9 0.9
22-
- TOM20
1 0.9 0.9 0.8
64-
- Lamin A
C
Ctrl
CCCP
Red: TOM20; Green: Flag; Blue: DAPI
D
shCtrl
shBRCA1-3
Oligomycin
kDa
0 15 30 60 120 240
0 15 30 60 120 240 min
250-
- BRCA1
1 1.1 1.2 1.2 0.9 0.9 0.6 0.6 0.5 0.5 0.4 0.3
148-
- p-Raptor
1 1.9 4.8 3.0 3.3 3.1 1 0.7 2.8 2.2 1.9 2.1
148-
- Raptor
1 1 1 0.8 0.8 0.8 0.8 1 1 1 1 1
64-
- p-AMPKα
1 1.9 2.3 2.1 1.8 1.5 0.4 0.6 1.4 1.1 0.8 0.4
64-
- AMPKα
1 1 1 1.1 1.2 0.9 1 1 1.1 1.1 1.2 1.1
36-
- β-actin
1 0.9 0.8 0.9 0.9 0.9 0.9 0.8 0.9 0.9 0.9 0.9
F
shCtrl
shBRCA1-3
- BRCA1
1 0.5
- β-actin
1 1
E
Ad-GFP
Ad-Cre
AICAR - + - +
- p-ACC
1 3.3 1.8 3.8
- ACC
1 0.8 0.8 0.8
- p-AMPKα
1 4.6 1 5.5
- AMPKα
1 0.8 1.4 0.8
- p-MFF
1 2.3 1.2 2.1
- MFF
1 1 1.1 1
- β-actin
1 0.9 1 1.1
G
shCtrl
shBRCA1-3
0 min
25 min
55 min
H
shCtrl
shDRP1
CCCP 0 6 12 24 0 6 12 24 h
kDa
64-
- DRP1
1 1 1.1 1.2 0.4 0.4 0.3 0.3
64-
- HSP60
1 0.7 0.5 0.4 0.9 1.1 1 1
22-
- COXII
1 0.7 0.3 0.1 0.8 0.7 0.4 0.3
16-
- LC3I
- LC3II
1 1.1 2.7 2.9 0.9 2.8 5.1 4.8
36-
- β-actin
1 1.1 1.2 1.3 1.3 1.2 1.2 1.3

Figure S5. BRCA1 is required for stress-induced mitochondrial fission via mediating AMPK activation. A) Immunoblot analysis of Flag-BRCA1 in Flag-tagged BRCA1 (Flag-BRCA1) MEFs compared with wildtype (WT) MEFs. **B)** Immunoblot analysis of BRCA1 level on mitochondria in Flag-BRCA1 MEFs under CCCP (30 μM) treatment. TOM20 and Lamin A are loading control for mitochondrial and nuclear protein, respectively. **C)** Representative images of BRCA1 in Flag-BRCA1 MEFs stained with Flag antibody. The cells were treated with or not CCCP. Scale bar, 50 μm. **D)** BRCA1 is required for Oligomycin-induced AMPK activation in 293T cells. shCtrl and shBRCA1 293T cells were treated by Oligomycin (10 μM) for indicated time points. **E)** BRCA1 has no effect on AICAR-induced AMPK activation. *Brca1*$^{flox/flox}$ MEFs infected with AdGFP or AdCre were treated with AICAR (2.5 mM) for 1 h. **F, G)** BRCA1 deficiency impairs CCCP-induced mitochondrial fission. (F) The BRCA1 levels in shCtrl and shBRCA1 Hela-HA-Parkin; (G) Mitochondrial morphology of shCtrl and shBRCA1 Hela-HA-Parkin with or without CCCP treatment for indicated time points. Mitochondria were marked by MitoDsRed. Arrows indicate mitochondrial morphology. **H)** Loss of DRP1 blocks CCCP-induced mitophagy. shCtrl and shDRP1 Hela-mCherryParkin were treated with CCCP for indicated time points. Data are representative of at least two independent experiments.

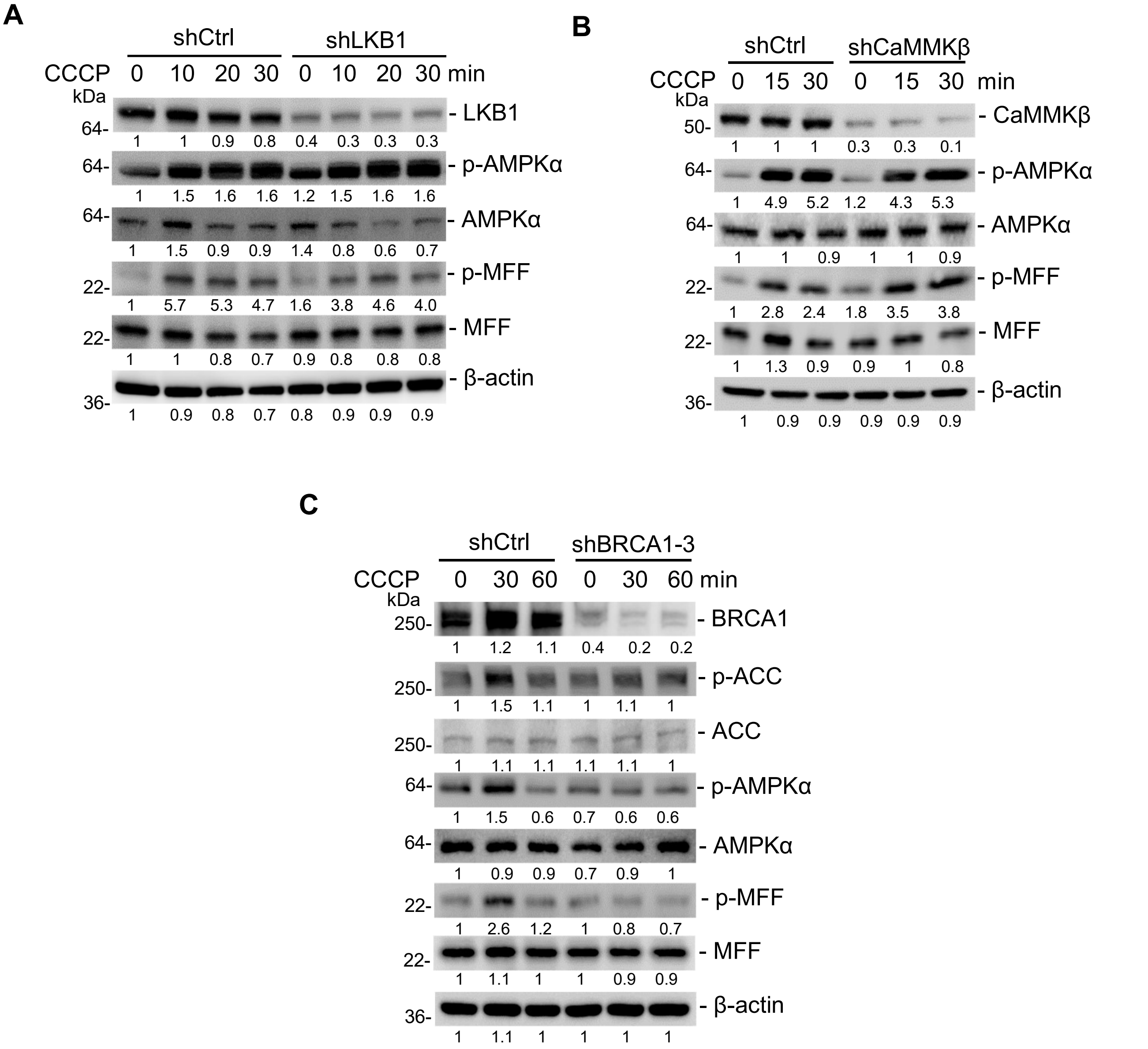

Figure S6. CCCP-induced AMPK activation is independent on LKB1 or CaMMKβ. A) LKB1 KD has no effect on CCCP-induced AMPK activation. shCtrl and shLKB1 293T cells were treated by CCCP for indicated time points. **B)** CaMMKβ KD has no effect on CCCP-induced AMPK activation. shCtrl and shCaMMKβ 293T cells were treated by CCCP for indicated time points. **C)** Immunoblot analysis of AMPK activation in shCtrl and shBRCA1 Hela cells that lack of LKB1. Data are representative of at least two independent experiments.

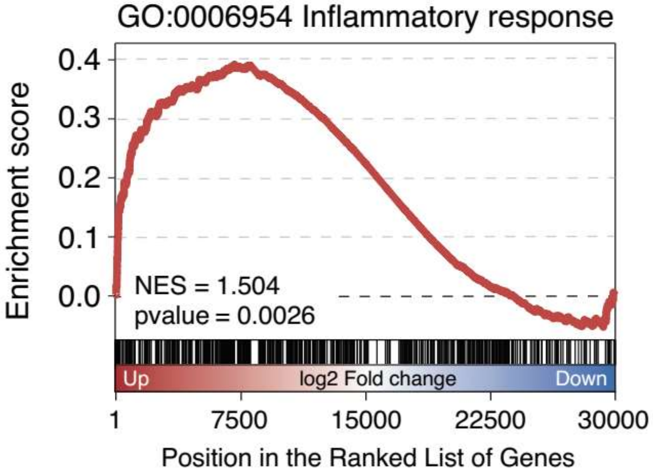

Figure S7. Inflammatory response significantly increases in *Brca1* mutant mammary gland. GSEA plot of enrichment in "inflammatory response" gene set upregulated in *Brca1* MT mammary glands.

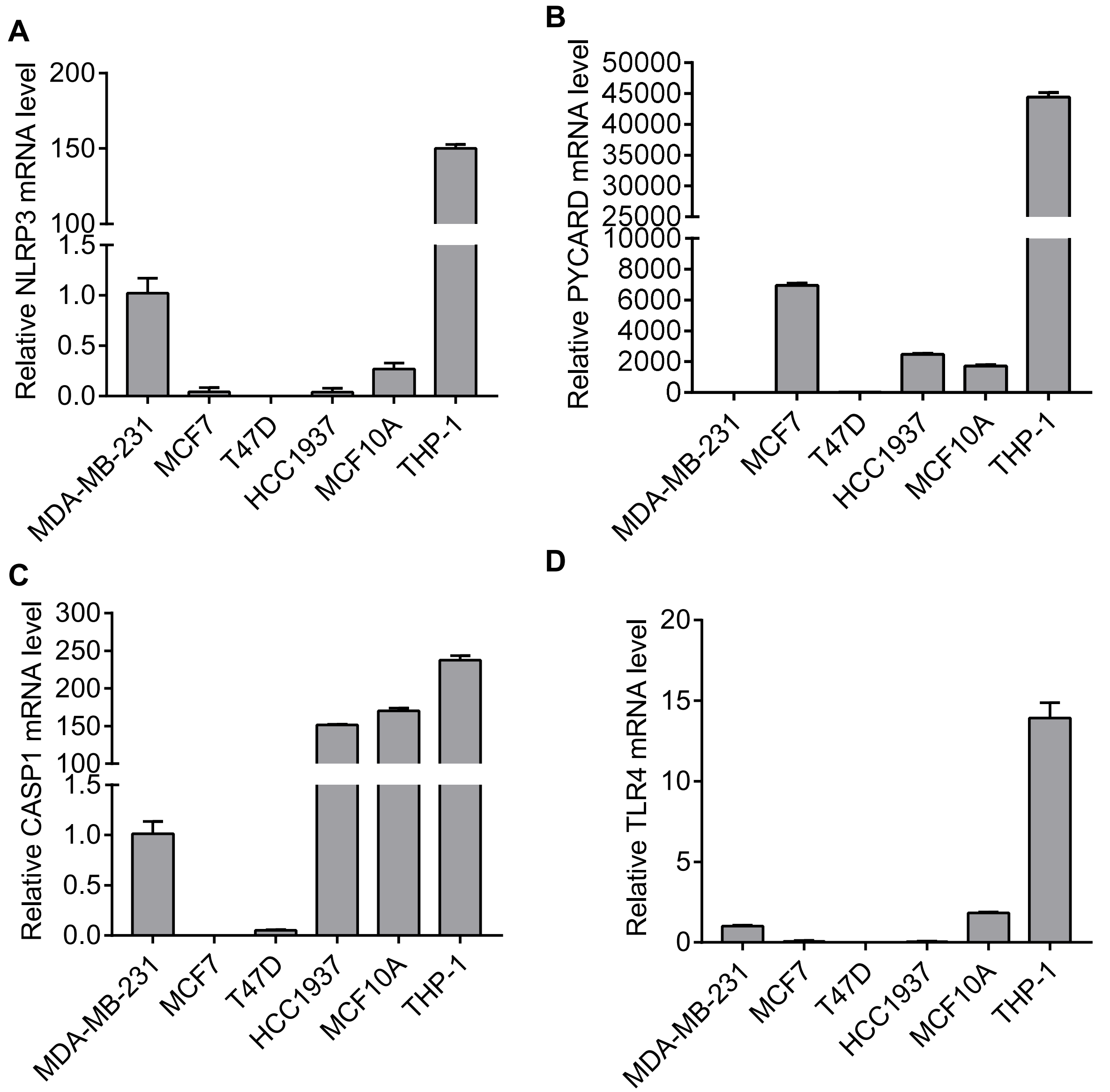

Figure S8. The expression of inflammasome associated genes in different cell lines. A) NLRP3 mRNA levels. **B)** PYCARD mRNA levels. **C)** CASP1 mRNA levels. **D)** TLR4 mRNA levels.

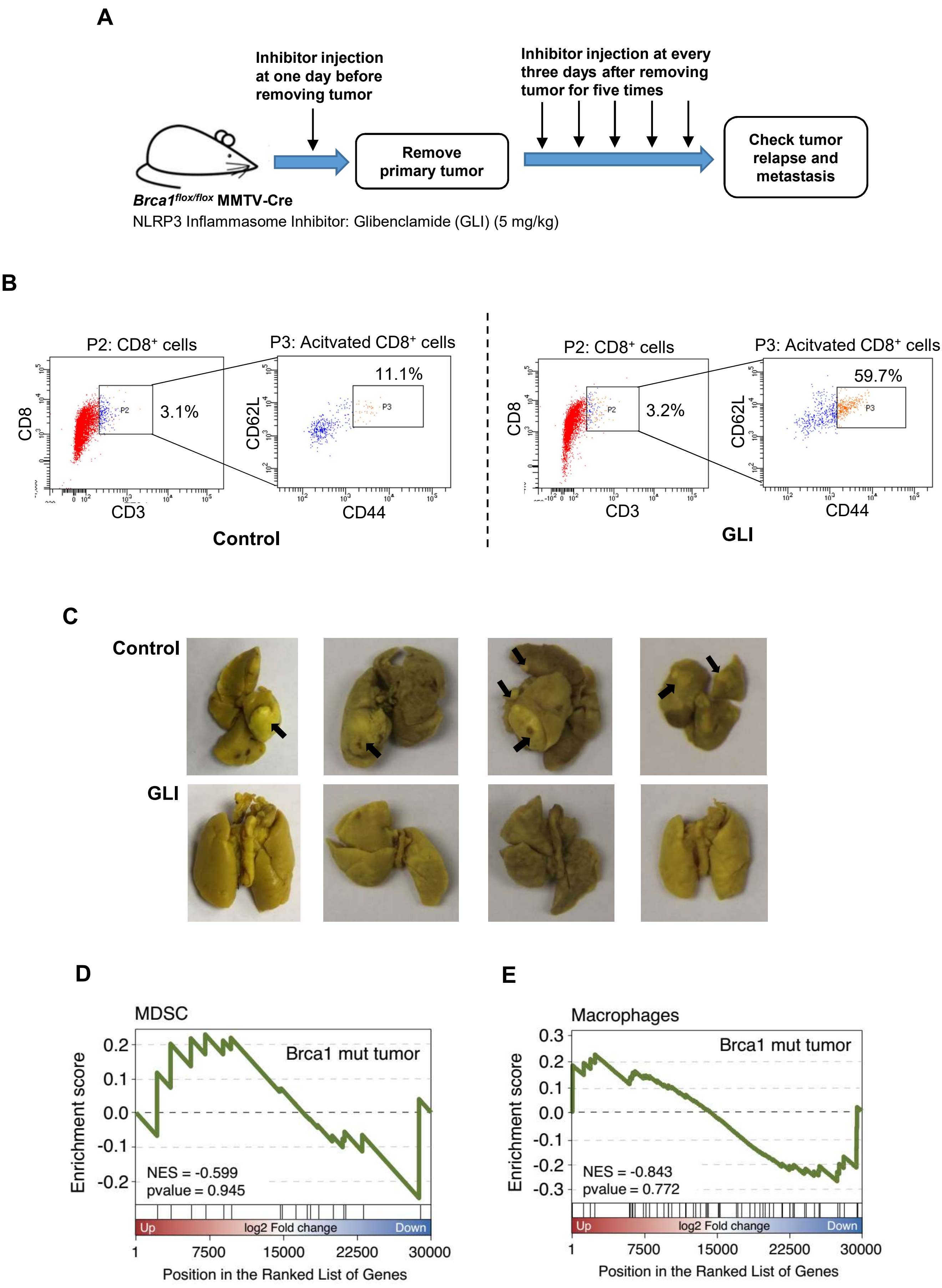

A
Inhibitor injection at one day before removing tumor
Inhibitor injection at every three days after removing tumor for five times
Remove primary tumor
Check tumor relapse and metastasis
Brca1flox/flox MMTV-Cre
NLRP3 Inflammasome Inhibitor: Glibenclamide (GLI) (5 mg/kg)
B
P2: CD8+ cells
P3: Acitvated CD8+ cells
3.1%
11.1%
CD8
CD3
CD62L
CD44
Control
P2: CD8+ cells
P3: Acitvated CD8+ cells
3.2%
59.7%
GLI
C
Control
GLI
D
MDSC
Brca1 mut tumor
NES = -0.599
pvalue = 0.945
Enrichment score
Up
log2 Fold change
Down
Position in the Ranked List of Genes
E
Macrophages
Brca1 mut tumor
NES = -0.843
pvalue = 0.772
Enrichment score
Position in the Ranked List of Genes

Figure S9. Inhibition of inflammasome activation attenuates *Brca1* mutant mammary tumor recurrence and metastasis. **A)** The flowchart of animal experiments. *Brca1*$^{flox/flox}$ MMTV-Cre mice developing mammary tumors (diameter around 1 cm) were i.p. injected with PBS or NLRP3 inflammasome inhibitor Glibenclamide (GLI, 5 mg/kg) one day before primary tumor removal. Then, the mice were administrated with PBS or GLI by i.p. continuous injection every three days for five times. **B)** Flow cytometric gating strategy for CD8^{+} T cell analysis. **C)** Representative images of lung metastasis of tumors after primary tumor removal. Arrows indicate metastatic tumors on lung. **D)** GSEA plot of enrichment in “MDSC” gene set has no significant difference in B.T versus Trp53.T. **E)** GSEA plot of enrichment in “Macrophages” gene set has no significant difference in B.T versus Trp53.T.
