# Supplementary material for: BRCA1 Deficiency Impairs Mitophagy and Promotes Inflammasome Activation and Mammary Tumor Metastasis
Source: Adv Sci (Weinh). 2020 Feb 14;7(6):1903616. doi: 10.1002/advs.201903616 (PMC7080549; doi:10.1002/advs.201903616)
Supplement: Supplementary file 4 — Supplemental Table 1 [file ADVS-7-1903616-s004.pdf]

Copyright WILEY-VCH Verlag GmbH & Co. KGaA, 69469 Weinheim, Germany, 2020.

ADVANCED SCIENCE Open Access

Supporting Information

for *Adv. Sci.,* DOI: 10.1002/advs.201903616

BRCA1 Deficiency Impairs Mitophagy and Promotes Inflammasome Activation and Mammary Tumor Metastasis

Qiang Chen, Josh Haipeng Lei, Jiaolin Bao, Haitao Wang, Wenhui Hao, Licen Li, Cheng Peng, Takaaki Masuda, Kai Miao, Jun Xu, Xiaoling Xu, and Chu-Xia Deng**

Table S1. The proteins pulled down with BRCA1

	Protein	Total	Unique	Accession
1	Glycogen phosphorylase, liver form OS=Homo sapiens GN=PYGL PE=1 SV=4	82	42	P06737
2	Desmoplakin OS=Homo sapiens GN=DSP PE=1 SV=3	45	29	P15924
3	78 kDa glucose-regulated protein OS=Homo sapiens GN=HSPA5 PE=1 SV=2	32	22	P11021
4	Stress-70 protein, mitochondrial OS=Homo sapiens GN=HSPA9 PE=1 SV=2	29	21	P38646
5	Clathrin heavy chain 1 OS=Homo sapiens GN=CLTC PE=1 SV=5	23	20	Q00610
6	Glycogen phosphorylase, brain form OS=Homo sapiens GN=PYGB PE=1 SV=5	33	18	P11216
7	Phosphorylase b kinase regulatory subunit alpha, liver isoform OS=Homo sapiens GN=PHKA2 PE=1 SV=1	20	18	P46019
8	Heat shock cognate 71 kDa protein OS=Homo sapiens GN=HSPA8 PE=1 SV=1	34	17	P11142
9	Heat shock 70 kDa protein 1A/1B OS=Homo sapiens GN=HSPA1A PE=1 SV=5	27	16	P08107
10	Nucleolin OS=Homo sapiens GN=NCL PE=1 SV=3	25	15	P19338
11	Filamin-A OS=Homo sapiens GN=FLNA PE=1 SV=4	14	14	P21333
12	Desmoglein-1 OS=Homo sapiens GN=DSG1 PE=1 SV=2	21	13	Q02413
13	Bifunctional glutamate/proline--tRNA ligase OS=Homo sapiens GN=EPRS PE=1 SV=5	14	13	P07814
14	Hornerin OS=Homo sapiens GN=HRNR PE=1 SV=2	36	12	Q86YZ3
15	Heat shock 70 kDa protein 1-like OS=Homo sapiens GN=HSPA1L PE=1 SV=2	24	12	P34931
16	Phosphorylase b kinase regulatory subunit beta OS=Homo sapiens GN=PHKB PE=1 SV=3	18	12	Q93100
17	1,4-alpha-glucan-branching enzyme OS=Homo sapiens GN=GBE1 PE=1 SV=3	17	12	Q04446
18	Polyadenylate-binding protein 1 OS=Homo sapiens GN=PABPC1 PE=1 SV=2	16	12	P11940
19	Nucleolar RNA helicase 2 OS=Homo sapiens GN=DDX21 PE=1 SV=5	14	12	Q9NR30
20	Leucine--tRNA ligase, cytoplasmic OS=Homo sapiens GN=LARS PE=1 SV=2	12	12	Q9P2J5
21	Tubulin alpha-1A chain OS=Homo sapiens GN=TUBA1A PE=1 SV=1	20	11	Q71U36
22	Heat shock protein 105 kDa OS=Homo sapiens GN=HSPH1 PE=1 SV=1	12	11	Q92598
23	Isoleucine--tRNA ligase, cytoplasmic OS=Homo sapiens GN=IARS PE=1 SV=2	12	11	P41252
24	Glycogen debranching enzyme OS=Homo sapiens GN=AGL PE=1 SV=3	11	11	P35573
25	Titin OS=Homo sapiens GN=TTN PE=1 SV=4	26	10	Q8WZ42
26	Filaggrin-2 OS=Homo sapiens GN=FLG2 PE=1 SV=1	21	10	Q5D862
27	Tubulin beta-2A chain OS=Homo sapiens GN=TUBB2A	14	10	Q13885

	PE=1 SV=1			
28	Heterogeneous nuclear ribonucleoprotein M OS=Homo sapiens GN=HNRNPM PE=1 SV=3	13	10	P52272
29	Heat shock 70 kDa protein 4 OS=Homo sapiens GN=HSPA4 PE=1 SV=4	10	10	P34932
30	T-complex protein 1 subunit theta OS=Homo sapiens GN=CCT8 PE=1 SV=4	10	10	P50990
31	Caspase-14 OS=Homo sapiens GN=CASP14 PE=1 SV=2	15	9	P31944
32	Cytoplasmic dynein 1 heavy chain 1 OS=Homo sapiens GN=DYNC1H1 PE=1 SV=5	11	9	Q14204
33	Aspartate--tRNA ligase, cytoplasmic OS=Homo sapiens GN=DARS PE=1 SV=2	10	9	P14868
34	CAD protein OS=Homo sapiens GN=CAD PE=1 SV=3	9	9	P27708
35	Spectrin alpha chain, non-erythrocytic 1 OS=Homo sapiens GN=SPTAN1 PE=1 SV=3	9	9	Q13813
36	Junction plakoglobin OS=Homo sapiens GN=JUP PE=1 SV=3	19	8	P14923
37	5'-AMP-activated protein kinase catalytic subunit alpha-1 OS=Homo sapiens GN=PRKAA1 PE=1 SV=4	11	8	Q13131
38	Glutamine--tRNA ligase OS=Homo sapiens GN=QARS PE=1 SV=1	9	8	P47897
39	RuvB-like 2 OS=Homo sapiens GN=RUVBL2 PE=1 SV=3	8	8	Q9Y230
40	Phosphorylase b kinase regulatory subunit alpha, skeletal muscle isoform OS=Homo sapiens GN=PHKA1 PE=1 SV=2	8	8	P46020
41	Phosphorylase b kinase gamma catalytic chain, testis/liver isoform OS=Homo sapiens GN=PHKG2 PE=1 SV=1	10	7	P15735
42	Protein disulfide-isomerase A6 OS=Homo sapiens GN=PDIA6 PE=1 SV=1	9	7	Q15084
43	T-complex protein 1 subunit alpha OS=Homo sapiens GN=TCP1 PE=1 SV=1	8	7	P17987
44	X-ray repair cross-complementing protein 5 OS=Homo sapiens GN=XRCC5 PE=1 SV=3	8	7	P13010
45	T-complex protein 1 subunit delta OS=Homo sapiens GN=CCT4 PE=1 SV=4	7	7	P50991
46	Lupus La protein OS=Homo sapiens GN=SSB PE=1 SV=2	7	7	P05455
47	X-ray repair cross-complementing protein 6 OS=Homo sapiens GN=XRCC6 PE=1 SV=2	7	7	P12956
48	U5 small nuclear ribonucleoprotein 200 kDa helicase OS=Homo sapiens GN=SNRNP200 PE=1 SV=2	7	7	O75643
49	Arginase-1 OS=Homo sapiens GN=ARG1 PE=1 SV=2	9	6	P05089
50	Elongation factor 1-alpha 1 OS=Homo sapiens GN=EEF1A1 PE=1 SV=1	9	6	P68104
51	Heat shock-related 70 kDa protein 2 OS=Homo sapiens GN=HSPA2 PE=1 SV=1	9	6	P54652
52	Heterogeneous nuclear ribonucleoprotein U OS=Homo sapiens GN=HNRNPU PE=1 SV=6	8	6	Q00839
53	Vimentin OS=Homo sapiens GN=VIM PE=1 SV=4	7	6	P08670
54	5'-AMP-activated protein kinase catalytic subunit alpha-2 OS=Homo sapiens GN=PRKAA2 PE=1 SV=2	7	6	P54646

55	Methionine--tRNA ligase, cytoplasmic OS=Homo sapiens GN=MARS PE=1 SV=2	7	6	P56192
56	Lamin-B1 OS=Homo sapiens GN=LMNB1 PE=1 SV=2	6	6	P20700
57	T-complex protein 1 subunit gamma OS=Homo sapiens GN=CCT3 PE=1 SV=4	6	6	P49368
58	60 kDa heat shock protein, mitochondrial OS=Homo sapiens GN=HSPD1 PE=1 SV=2	6	6	P10809
59	Splicing factor 3B subunit 3 OS=Homo sapiens GN=SF3B3 PE=1 SV=4	6	6	Q15393
60	ATP synthase subunit beta, mitochondrial OS=Homo sapiens GN=ATP5B PE=1 SV=3	6	6	P06576
61	Filamin-B OS=Homo sapiens GN=FLNB PE=1 SV=2	6	6	O75369
62	Tubulin beta-1 chain OS=Homo sapiens GN=TUBB1 PE=1 SV=1	9	5	Q9H4B7
63	Tubulin beta-3 chain OS=Homo sapiens GN=TUBB3 PE=1 SV=2	7	5	Q13509
64	Annexin A2 OS=Homo sapiens GN=ANXA2 PE=1 SV=2	7	5	P07355
65	Heterogeneous nuclear ribonucleoprotein L OS=Homo sapiens GN=HNRNPL PE=1 SV=2	7	5	P14866
66	Actin, aortic smooth muscle OS=Homo sapiens GN=ACTA2 PE=1 SV=1	7	5	P62736
67	Myosin-10 OS=Homo sapiens GN=MYH10 PE=1 SV=3	7	5	P35580
68	Putative pre-mRNA-splicing factor ATP-dependent RNA helicase DHX15 OS=Homo sapiens GN=DHX15 PE=1 SV=2	6	5	O43143
69	Lysine--tRNA ligase OS=Homo sapiens GN=KARS PE=1 SV=3	6	5	Q15046
70	Catalase OS=Homo sapiens GN=CAT PE=1 SV=3	5	5	P04040
71	Heat shock 70 kDa protein 4L OS=Homo sapiens GN=HSPA4L PE=1 SV=3	5	5	O95757
72	Elongation factor 1-gamma OS=Homo sapiens GN=EEF1G PE=1 SV=3	5	5	P26641
73	Myosin-9 OS=Homo sapiens GN=MYH9 PE=1 SV=4	5	5	P35579
74	Nucleoprotein TPR OS=Homo sapiens GN=TPR PE=1 SV=3	5	5	P12270
