# Supplementary material for: BRCA1 Deficiency Impairs Mitophagy and Promotes Inflammasome Activation and Mammary Tumor Metastasis
Source: Adv Sci (Weinh). 2020 Feb 14;7(6):1903616. doi: 10.1002/advs.201903616 (PMC7080549; doi:10.1002/advs.201903616)
Supplement: Supplementary file 5 — Supplemental Table 2 [file ADVS-7-1903616-s005.pdf]

Copyright WILEY-VCH Verlag GmbH & Co. KGaA, 69469 Weinheim, Germany, 2020.

ADVANCED SCIENCE
Open Access

Supporting Information

for *Adv. Sci.,* DOI: 10.1002/advs.201903616

BRCA1 Deficiency Impairs Mitophagy and Promotes Inflammasome Activation and Mammary Tumor Metastasis

Qiang Chen, Josh Haipeng Lei, Jiaolin Bao, Haitao Wang, Wenhui Hao, Licen Li, Cheng Peng, Takaaki Masuda, Kai Miao, Jun Xu, Xiaoling Xu, and Chu-Xia Deng**

Table S2. Antibodies used for experiments

WB Antibody	Source	Identifier
COXII	Abcam	Cat# ab110258; RRID:AB_10887758
BRCA1	Santa Cruz Biotechnology	Cat# sc-642; RRID:AB_630944
COXIV	Cell Signaling Technology	Cat# 4844; RRID:AB_2085427
TOM20	Santa Cruz Biotechnology	Cat# sc-11415; RRID:AB_2207533
p62	MBL International	Cat# M162-3; RRID:AB_1279299
LC3B	Cell Signaling Technology	Cat# 3868; RRID:AB_2137707
MFN1	Abcam	Cat# ab57602; RRID:AB_2142624
MFN1	Cell Signaling Technology	Cat# 14739; RRID:AB_2744531
MFN2	Abcam	Cat# ab56889; RRID:AB_2142629
MFN2	Cell Signaling Technology	Cat# 11925; RRID:AB_2750893
OPA1	Abcam	Cat# ab42364; RRID:AB_944549
DRP1	Abcam	Cat# ab56788; RRID:AB_941306
FIS1	Santa Cruz Biotechnology	Cat# sc-98900; RRID:AB_2246809
Flag	Sigma	Cat# F1804; RRID:AB_262044
Lamin A/C	Santa Cruz Biotechnology	Cat# sc-6215; RRID:AB_648152
AMPKα	Cell Signaling Technology	Cat# 2532; RRID:AB_330331
AMPKα	Cell Signaling Technology	Cat# 2793; RRID:AB_915794
p-ACC (Ser79)	Cell Signaling Technology	Cat# 3661; RRID:AB_330337
ACC	Cell Signaling Technology	Cat# 3662; RRID:AB_2219400
p-Raptor (Ser792)	Cell Signaling Technology	Cat# 2083; RRID:AB_2249475
Raptor	Cell Signaling Technology	Cat# 2280; RRID:AB_561245
p-AMPKα (Thr172)	Cell Signaling Technology	Cat# 2535; RRID:AB_331250
p-MFF (Ser146)	Cell Signaling Technology	Cat# 49281; RRID:AB_2799354
MFF	Cell Signaling Technology	Cat# 86668; RRID:AB_2734126
p-ATM (S1981)	Abcam	Cat# ab81292; RRID:AB_1640207
ATM	Abcam	Cat# ab78; RRID:AB_306089
IL-1β	Cell Signaling Technology	Cat# 12703; RRID:AB_2737350
Cleaved-IL-1β (Asp116)	Cell Signaling Technology	Cat# 83186; RRID:AB_2800010
Caspase-1	Cell Signaling Technology	Cat# 3866; RRID:AB_2069051
Cleaved Caspase-1 (Asp297)	Cell Signaling Technology	Cat# 4199; RRID:AB_1903916
Caspase-1 (p20) (mouse)	AdipoGen Life Sciences	Cat# AG-20B-0042-C100; RRID:AB_2755041
PINK1	Cell Signaling Technology	Cat# 6946; RRID:AB_1117906
Ubiquitin	Sigma	Cat# SAB1306582; N/A
HSP60	Santa Cruz Biotechnology	Cat# sc-59567; RRID:AB_783870
LKB1	Santa Cruz Biotechnology	Cat# sc-374334; RRID:AB_10989381

CaMMKβ	Santa Cruz Biotechnology	Cat# sc-271674; RRID:AB_10708844
β-actin	Sigma	Cat# A5316; RRID:AB_476743
α-Tubulin	Sigma	Cat# T5168; RRID:AB_477579

**IP Antibodies**	**Source**	**Catalog #**
BRCA1	Abcam	Cat# ab16780; RRID:AB_2259338
AMPKα1	Abcam	Cat# ab32047; RRID:AB_722764
AMPKα2	Abcam	Cat# ab3760; RRID:AB_304055
MBP	Santa Cruz Biotechnology	Cat# sc-808; RRID:AB_675709

**IF Antibodies**	**Source**	**Catalog #**
ATP5B	EMD Millipore	Cat# MAB3494; RRID:AB_177597
BRCA1	EMD Millipore	Cat# 07-434; RRID:AB_2275035
TIM50	Santa Cruz Biotechnology	Cat# sc-393678; RRID:AB_2714191
F4/80	ThermoFisher Scientific	Cat# 14-4801-81; RRID:AB_467557
Drp1	Abcam	Cat# ab56788; RRID:AB_941306
DNA	Progen	Cat# 61014; RRID:AB_2750935
Flag	Sigma	Cat# F1804; RRID:AB_262044

**Flow cytometry Antibodies**	**Source**	**Catalog #**
CD3e Monoclonal Antibody (145-2C11), Alexa Fluor 488	ThermoFisher Scientific	Cat# 53-0031-82; RRID:AB_469889
CD8a Monoclonal Antibody (53-6.7), PerCP-eFluor 710	ThermoFisher Scientific	Cat# 46-0081-82; RRID:AB_1834433
CD44 Monoclonal Antibody (IM7), PE	ThermoFisher Scientific	Cat# 12-0441-82; RRID:AB_465664
CD62L (L-Selectin) Monoclonal Antibody (MEL-14), eFluor 450	ThermoFisher Scientific	Cat# 48-0621-82; RRID:AB_1963590
