# Supplementary material for: BRCA1 Deficiency Impairs Mitophagy and Promotes Inflammasome Activation and Mammary Tumor Metastasis
Source: Adv Sci (Weinh). 2020 Feb 14;7(6):1903616. doi: 10.1002/advs.201903616 (PMC7080549; doi:10.1002/advs.201903616)
Supplement: Supplementary file 6 — Supplemental Table 3 [file ADVS-7-1903616-s006.pdf]

Copyright WILEY-VCH Verlag GmbH & Co. KGaA, 69469 Weinheim, Germany, 2020.

Supporting Information

for *Adv. Sci.,* DOI: 10.1002/advs.201903616

BRCA1 Deficiency Impairs Mitophagy and Promotes Inflammasome Activation and Mammary Tumor Metastasis

Qiang Chen, Josh Haipeng Lei, Jiaolin Bao, Haitao Wang, Wenhui Hao, Licen Li, Cheng Peng, Takaaki Masuda, Kai Miao, Jun Xu, Xiaoling Xu, and Chu-Xia Deng**

Table S3. Primers used for experiments

Target	Species	sequence 5'-3'
**BRCA1 F**	Mouse	GGAAATGGCAACTTGCCTAG
**BRCA1 R**	Mouse	CTGCGAGCAGTCTTCAGAAAG
**CD206 F**	Mouse	CTCTGTTCAGCTATTGGACGC
**CD206 R**	Mouse	CGGAATTTCTGGGATTCAGCTTC
**CD163 F**	Mouse	ATGGGTGGACACAGAATGGTT
**CD163 R**	Mouse	CAGGAGCGTTAGTGACAGCAG
**MFN1 F**	Mouse	TCTCCAAGCCCAACATCTTC
**MFN1 R**	Mouse	GAGCTTCCGACGGACTTACA
**MFN2 F**	Mouse	GTCCTGGACGTCAAAGGGTA
**MFN2 R**	Mouse	CAATCCCAGATGGCAGAACT
**Mgl2 F**	Mouse	AGGCAGCTGCTATTGGTTCTCTGA
**Mgl2 R**	Mouse	AGTTGACCACCACCAGGTGAGAAT
**MFN1 F**	Human	GGCACTTGCTGAAGGATTTC
**MFN1 R**	Human	TCAGCTGCCAGGTTTACTGA
**MFN2 F**	Human	GCACTTCTTCCACAAGGTGA
**MFN2 R**	Human	AGCTCATCCACCAGGAAGC
**NLRP3 F**	Human	GATCTTCGCTGCGATCAACAG
**NLRP3 R**	Human	CGTGCATTATCTGAACCCCAC
**PYCARD F**	Human	TGGATGCTCTGTACGGGAAG
**PYCARD R**	Human	CCAGGCTGGTGTGAAACTGAA
**CASP1 F**	Human	TTTCCGCAAGGTTCGATTTTCA
**CASP1 R**	Human	GGCATCTGCGCTCTACCATC
**TLR4 F**	Human	TTTGGACAGTTTCCCACATTGA
**TLR4 R**	Human	AAGCATTCCCACCTTTGTTGG
**18S F**	Human/Mouse	AGTCCCTGCCCTTTGTACACA
**18S R**	Human/Mouse	CGATCCGAGGGCCTCACTA
