# Supplementary material for: BRCA1 Deficiency Impairs Mitophagy and Promotes Inflammasome Activation and Mammary Tumor Metastasis
Source: Adv Sci (Weinh). 2020 Feb 14;7(6):1903616. doi: 10.1002/advs.201903616 (PMC7080549; doi:10.1002/advs.201903616)
Supplement: Supplementary file 7 — Supplemental Table 4 [file ADVS-7-1903616-s007.pdf]

Copyright WILEY-VCH Verlag GmbH & Co. KGaA, 69469 Weinheim, Germany, 2020.

ADVANCED SCIENCE
Open Access

Supporting Information

for *Adv. Sci.,* DOI: 10.1002/advs.201903616

BRCA1 Deficiency Impairs Mitophagy and Promotes Inflammasome Activation and Mammary Tumor Metastasis

Qiang Chen, Josh Haipeng Lei, Jiaolin Bao, Haitao Wang, Wenhui Hao, Licen Li, Cheng Peng, Takaaki Masuda, Kai Miao, Jun Xu, Xiaoling Xu, and Chu-Xia Deng**

Table S4. Information of clinical data from METABRIC

***BRCA1* mutant breast cancer patients**

	Case ID	Protein Change	Mutation Type	Cancer Type
1	MB.0346	R1737T	Missense_Mutation	Breast Invasive Ductal Carcinoma
2	MB.0420	V1654Cfs*4	Frame_Shift_Del	Breast Invasive Ductal Carcinoma
3	MB.0532	Y1127H	Missense_Mutation	Breast Invasive Ductal Carcinoma
4	MB.0320	D420G	Missense_Mutation	Breast Invasive Lobular Carcinoma
5	MB.4627	S153R	Missense_Mutation	Breast Mixed Ductal and Lobular Carcinoma
6	MB.4674	G1087E	Missense_Mutation	Breast Invasive Ductal Carcinoma
7	MB.5152	M1827L	Missense_Mutation	Breast Invasive Ductal Carcinoma
8	MB.5323	E880Rfs*13	Frame_Shift_Del	Breast Invasive Ductal Carcinoma
9	MB.5232	X1453_splice	Splice_Site	Breast Invasive Ductal Carcinoma
10	MB.5088	I946V	Missense_Mutation	Breast Invasive Ductal Carcinoma
11	MB.5107	E1494Kfs*11	Frame_Shift_Del	Breast Invasive Lobular Carcinoma
12	MB.5070	T150Pfs*13	Frame_Shift_Del	Breast Invasive Ductal Carcinoma
13	MB.5062	L1439F	Missense_Mutation	Breast Invasive Ductal Carcinoma
14	MB.5166	R613K	Missense_Mutation	Breast Invasive Ductal Carcinoma
15	MB.5602	E1836K	Missense_Mutation	Breast Invasive Ductal Carcinoma
16	MB.2735	W372R	Missense_Mutation	Breast Invasive Ductal Carcinoma
17	MB.2617	Y1127H	Missense_Mutation	Breast Invasive Ductal Carcinoma
18	MB.2922	I216S	Missense_Mutation	Breast Invasive Ductal Carcinoma
19	MB.4828	C305S	Missense_Mutation	Breast Invasive Lobular Carcinoma
20	MB.4660	X1760_splice	Splice_Site	Breast Invasive Ductal Carcinoma
21	MB.6248	X1663_splice	Splice_Site	Breast Invasive Ductal Carcinoma
22	MB.6098	D1692N	Missense_Mutation	Breast Invasive Ductal Carcinoma
23	MB.6047	E1287K	Missense_Mutation	Breast Invasive Ductal Carcinoma
24	MB.6271	L502Sfs*2	Frame_Shift_Del	Breast Invasive Lobular Carcinoma
25	MB.5428	S1266T	Missense_Mutation	Breast Invasive Ductal Carcinoma
26	MB.6060	S282*	Nonsense_Mutation	Breast Invasive Ductal Carcinoma
27	MB.6178	V452A	Missense_Mutation	Breast Invasive Ductal Carcinoma
28	MB.6062	I171M	Missense_Mutation	Invasive Breast Carcinoma
29	MB.5164	E143K	Missense_Mutation	Breast Invasive Ductal Carcinoma
30	MB.7048	F709Sfs*29	Frame_Shift_Ins	Breast Invasive Ductal Carcinoma
31	MB.7038	Q202Kfs*32	Frame_Shift_Del	Breast Invasive Ductal Carcinoma
32	MB.7032	K1667Qfs*11	Frame_Shift_Ins	Breast Invasive Ductal Carcinoma
33	MB.7040	R1074T	Missense_Mutation	Breast Invasive Ductal Carcinoma
34	MB.0163	S1551C	Missense_Mutation	Breast Invasive Ductal Carcinoma
35	MB.0234	E914K	Missense_Mutation	Breast Invasive Lobular Carcinoma
36	MB.5465	D560Efs*6	Frame_Shift_Ins	Breast Invasive Ductal Carcinoma

***TP53* mutant breast cancer patients**

	Case ID	Protein Change	Cancer Type	Mutation Type
1	MB.0164	R175H	Missense_Mutation	Breast Invasive Ductal Carcinoma
2	MB.0102	R175H	Missense_Mutation	Breast Invasive Lobular Carcinoma
3	MB.0516	R175H	Missense_Mutation	Breast Invasive Ductal Carcinoma
4	MB.0585	R175H	Missense_Mutation	Breast Invasive Ductal Carcinoma
5	MB.0115	R175H	Missense_Mutation	Breast Invasive Ductal Carcinoma
6	MB.0608	R175H	Missense_Mutation	Invasive Breast Carcinoma
7	MB.5259	R175H	Missense_Mutation	Breast Invasive Ductal Carcinoma
8	MB.5292	R175H	Missense_Mutation	Breast Invasive Ductal Carcinoma
9	MB.5560	R175H	Missense_Mutation	Breast Invasive Ductal Carcinoma
10	MB.5294	R175H	Missense_Mutation	Breast Invasive Ductal Carcinoma
11	MB.3067	R175H	Missense_Mutation	Invasive Breast Carcinoma
12	MB.0476	R175H	Missense_Mutation	Breast Invasive Ductal Carcinoma
13	MB.3383	R175H	Missense_Mutation	Breast Invasive Ductal Carcinoma
14	MB.7104	R175H	Missense_Mutation	Breast Invasive Ductal Carcinoma
15	MB.7158	R175H	Missense_Mutation	Breast Invasive Ductal Carcinoma
16	MB.7165	R175H	Missense_Mutation	Breast Invasive Ductal Carcinoma
17	MB.7150	R175H	Missense_Mutation	Breast Invasive Ductal Carcinoma
18	MB.2833	R175H	Missense_Mutation	Breast Invasive Ductal Carcinoma
19	MB.2645	R175H	Missense_Mutation	Breast Invasive Ductal Carcinoma
20	MB.4865	R175H	Missense_Mutation	Breast Invasive Ductal Carcinoma
21	MB.4860	R175H	Missense_Mutation	Breast Invasive Lobular Carcinoma
22	MB.4949	R175H	Missense_Mutation	Breast Invasive Ductal Carcinoma
23	MB.6010	R175H	Missense_Mutation	Invasive Breast Carcinoma
24	MB.4005	R175H	Missense_Mutation	Breast Invasive Lobular Carcinoma
25	MB.4270	R175H	Missense_Mutation	Breast Invasive Ductal Carcinoma
26	MB.4250	R175H	Missense_Mutation	Breast Invasive Ductal Carcinoma
27	MB.4333	R175H	Missense_Mutation	Breast Invasive Ductal Carcinoma
28	MB.6036	R175H	Missense_Mutation	Invasive Breast Carcinoma
29	MB.6144	R175H	Missense_Mutation	Invasive Breast Carcinoma
30	MB.6080	R175H	Missense_Mutation	Breast Invasive Ductal Carcinoma
31	MB.6237	R175H	Missense_Mutation	Breast Invasive Ductal Carcinoma
32	MB.0469	R175H	Missense_Mutation	Breast Invasive Ductal Carcinoma
33	MB.0149	R175H	Missense_Mutation	Breast Invasive Ductal Carcinoma
34	MB.0652	R175H	Missense_Mutation	Breast Invasive Ductal Carcinoma
