# Supplementary material for: BRCA1 Deficiency Impairs Mitophagy and Promotes Inflammasome Activation and Mammary Tumor Metastasis
Source: Adv Sci (Weinh). 2020 Feb 14;7(6):1903616. doi: 10.1002/advs.201903616 (PMC7080549; doi:10.1002/advs.201903616)
Supplement: Supplementary file 8 — Supplemental Table 5 [file ADVS-7-1903616-s008.pdf]

Copyright WILEY-VCH Verlag GmbH & Co. KGaA, 69469 Weinheim, Germany, 2020.

ADVANCED SCIENCE
Open Access

# Supporting Information

for *Adv. Sci.*, DOI: 10.1002/advs.201903616

# BRCA1 Deficiency Impairs Mitophagy and Promotes Inflammasome Activation and Mammary Tumor Metastasis

*Qiang Chen,* Josh Haipeng Lei, Jiaolin Bao, Haitao Wang, Wenhui Hao, Licen Li, Cheng Peng, Takaaki Masuda, Kai Miao, Jun Xu, Xiaoling Xu, and Chu-Xia Deng**

## Table S5. M1/M2 macrophage signature genes

**Mouse**

| No. | M1 marker genes | M2 marker genes |
|---|---|---|
| 1 | Cd38 | Ptgs1 |
| 2 | Cfb | Egr2 |
| 3 | Slfn4 | Olfm1 |
| 4 | H2-Q6 | Flrt2 |
| 5 | Fpr1 | P2ry1 |
| 6 | Slfn1 | Vwf |
| 7 | Gpr18 | Bcar3 |
| 8 | Ccrl2 | Il6st |
| 9 | Fpr2 | Tanc2 |
| 10 | Cxcl10 | Mmp12 |
| 11 | Mpa2l | Tcfec |
| 12 | Oasl1 | Clec7a |
| 13 | Tlr2 | Matk |
| 14 | Ms4a4c | Myc |
| 15 | LOC100503664 | Clec10a |
| 16 | Irak3 | Amz1 |
| 17 | Hp | Tmem158 |
| 18 | Itgal | Tiam1 |
| 19 | Herc6 | Rhoj |
| 20 | Cd300lf | Mmp9 |
| 21 | Isf20 | Mrc1 |
| 22 | Pstpip2 | Atp6v0a1 |
| 23 | Cp | Lmna |
| 24 | Isg15 | Chst7 |
| 25 | Ifi44 | Atp6v0d2 |
| 26 | E030037K03Rik | Gnb4 |
| 27 | Saa3 | Emp2 |
| 28 | Ifit1 | Cd300ld |
| 29 | Marco | Cd83 |
| 30 | F11r | Socs6 |
| 31 | Rsad2 | Actn1 |
| 32 | Ddx60 | Plk2 |
| 33 | Pilr1 | Ptpla |
| 34 | Cpd | |
| 35 | Fam26f | |
| 36 | Aoah | |
| 37 | Gngt2 | |
| 38 | Mx1 | |

| | |
|---|---|
| 39 | Pyhin1 |
| 40 | Epb4.1l3 |
| 41 | Slfn8 |
| 42 | Arhgap24 |
| 43 | Nfkbiz |
| 44 | Gbp6 |
| 45 | Stat1 |
| 46 | Zpb1 |
| 47 | D14Erd668e |
| 48 | Ddx58 |
| 49 | Tuba4a |
| 50 | H2-T10 |
| 51 | Ebi3 |
| 52 | Fam176b |
| 53 | Xaf1 |
| 54 | Stat2 |
| 55 | Sepx1 |
| 56 | Ifit2 |

**Human**

| No. | M1 marker genes | M2 marker genes |
|---|---|---|
| 1 | SLFN12L | EGR2 |
| 2 | CD38 | MATK |
| 3 | FPR1 | MYC |
| 4 | EBI3 | SOCS6 |
| 5 | NFKBIZ | P2RY1 |
| 6 | CP | TMEM158 |
| 7 | CALHM6 | PTGS1 |
| 8 | XAF1 | MMP12 |
| 9 | DDX58 | ATP6V0A1 |
| 10 | MARCO | OLFM1 |
| 11 | PSTPIP2 | RHOJ |
| 12 | GPR18 | ACTN1 |
| 13 | OASL | IL6ST |
| 14 | FPR2 | PLK2 |
| 15 | ITGAL | AMZ1 |
| 16 | IFIT2 | TANC2 |
| 17 | STAT2 | CD83 |
| 18 | AOAH | VWF |
| 19 | TUBA4A | FLRT2 |
| 20 | HP | CLEC7A |

| | | |
|---|---|---|
| 21 | ARHGAP24 | MRC1 |
| 22 | DDX60 | EMP2 |
| 23 | CD300LF | ATP6V0D2 |
| 24 | ISG15 | BCAR3 |
| 25 | TLR2 | CHST7 |
| 26 | IFI44 | TIAM1 |
| 27 | CFB | MMP9 |
| 28 | IRAK3 | LMNA |
| 29 | F11R | GNB4 |
| 30 | RSAD2 | |
| 31 | CCRL2 | |
| 32 | IFIT1B | |
| 33 | CXCL10 | |
| 34 | HERC6 | |
| 35 | STAT1 | |
| 36 | CPD | |
| 37 | GNGT2 | |
